# Oxidized Hemoglobin Is Antigenic and Immunogenic in Lupus

**DOI:** 10.3389/fimmu.2017.00732

**Published:** 2017-06-26

**Authors:** Sonia Jain, Anjali Bose, Banajit Bastia, Hritika Sharma, Ruchi Sachdeva, Arun K. Jain, Rahul Pal

**Affiliations:** ^1^Immunoendocrinology Laboratory, National Institute of Immunology, New Delhi, India; ^2^Division of Electron Microscopy, National Institute of Pathology—ICMR, New Delhi, India

**Keywords:** hemoglobin, antigen spreading, glomerulonephritis, lupus, systemic autoimmunity

## Abstract

Hemolysis-associated anemia is characteristic of diseases such as atherosclerosis, lupus, malaria, and leishmaniasis; the toxic effects of free hemoglobin (Hb) have been extensively described. This study was based on the premise that release of this sequestered, inflammatory molecule can result in deleterious immunological consequences, particularly in the context of pre-existing lupus. IgG anti-Hb responses were detected in the sera of lupus patients. Lupus-prone mice exhibited heightened plasma Hb levels, and ferric (Fe^3+^) Hb triggered preferential release of lupus-associated cytokines from splenocytes derived from aging lupus-prone mice. Anti-Hb B cell precursor frequencies were heightened in such mice, which also expressed increased titers of anti-Hb antibodies in serum and in kidney eluates. Fe^3+^ Hb preferentially increased the functional maturation of bone marrow-derived dendritic cells (BMDCs) from lupus-prone mice, effects abrogated upon the inhibition of Stat3. Hb interacted with lupus-associated autoantigens extruded during apoptosis and coincubation of Hb and apoptotic blebs had additional maturation-inducing effects on lupus BMDCs. Immunization with Hb in lupus-prone mice induced antigen spreading to lupus-associated moieties; Hb-interacting autoantigens were preferentially targeted and increased complement deposition and glomerulosclerosis were observed. Hb therefore demonstrates both antigenicity and immunogenicity and triggers specific immuno-pathological effects in a lupus milieu.

## Introduction

Hemolysis, which characterizes several diseases of both infectious and autoimmune etiology, results in the release of hemoglobin (Hb) into circulation. Normally, haptoglobin (Hp) binds free Hb and the Hp–Hb complex is cleared *via* CD163-mediated endocytosis (1). In many hemolytic diseases, Hb concentrations exceed Hp-binding capacity (2, 3). Ferrous (Fe^2+^) Hb has a tendency to undergo oxidation to ferric (Fe^3+^) Hb (also referred to as methemoglobin) and to ferryl (Fe^4+^) Hb and may also release heme (4), leading to the formation of ferryl protein radicals (^⋅^P-Fe^4+^) and hemichromes (5). Hb, its oxidized forms, and heme have all been shown to be toxic to various cells; the vasoactivity, redox activity, and pro-inflammatory effects of Hb are well documented (6–10).

An inflammatory synergy between Hb and other molecules has been demonstrated. For example, Hb can enhance the secretion of inflammatory cytokines induced by toll-like receptor (TLR) 2, TLR3, TLR4, TLR7, and TLR9 agonists (11). While Hb is known to bind LPS (a TLR4 ligand) and increase its biological activity (12), the mechanisms by which synergy between Hb and other TLR ligands is achieved are not known. Since endogenous TLR ligands, such as those for TLR7/8 and TLR9, have been implicated in systemic autoimmunity (13), the binding of Hb to such ligands could have physiological and immunological effects.

The release of previously sequestered Hb, under conditions already rendered inflammatory because of on-going autoimmune responses (as in lupus), could lead to a break in immunological tolerance toward the molecule, an event which could entail pathophysiological consequences. Scattered evidence does suggest propensity for the generation of anti-Hb autoimmune responses. For example, T cell reactivity against autologous Hb has been demonstrated in both non-autoimmune and autoimmune-prone mice (14). Interestingly, in a specific instance, tumor-directed T cells were described to dominantly recognize Hb-derived peptides (15). Antigen microarray analysis of cord blood has revealed the existence of antibodies against Hb (16), and anti-Hb antibodies have been described in autoimmune human and murine sera (17). Humoral anti-Hb autoimmune responses remain poorly characterized, however, and potential mechanisms contributing to, as well as the downstream consequences of, a break of immunological tolerance to Hb are currently unknown.

The current study was undertaken to elucidate both the antigenicity and immunogenicity of Hb and to evaluate its effects on innate and adaptive immune cells, specifically in the context of lupus.

## Materials and Methods

### Human Sera and Animals

This study was carried out in accordance with the recommendations of the ethical guidelines for biomedical research on human participants laid down by the Indian Council of Medical Research with written informed consent from all subjects. Patients on follow-up were females (aged between 23 and 45 years) of North Indian ethnicity. All subjects gave written informed consent in accordance with the Declaration of Helsinki. The protocol was approved by the Institutional Human Ethics Committee of the National Institute of Immunology.

This study was carried out in accordance with the recommendations of Committee for the Purpose of Control and Supervision of Experiments on Animals (CPCSEA). The protocol was approved by the Institutional Animal Ethics Committee (IAEC Number: 323/13) of the National Institute of Immunology. NZM2410 (hereafter referred to as NZM), NZB × NZW F1 (hereafter referred to as NZB/W F1), FVB and C57BL/6 mice were obtained from The Jackson Laboratory and maintained at the National Institute of Immunology, New Delhi. Female mice were used for all experiments.

### Anti-Hb Reactivity in Human Patients

Reactivity of antibodies in control sera (*n* = 28), SLE patients (*n* = 28), malaria (*P. falciparum*) patients (*n* = 10), rheumatoid arthritis patients (*n* = 20), and vitiligo patients (*n* = 20) to human Hb (hHb) (Sigma) was assessed by ELISA, using standard protocols; sera were diluted (1:250) before analysis. HRP-conjugated goat anti-human IgG + M antibodies (Jackson ImmunoResearch) were employed for detection. IgG immunoglobulins were purified from SLE sera containing antibodies reactive toward hHb (*n* = 8) and from control sera (*n* = 8) by Protein G affinity chromatography. Reactivity of purified IgG (1 μg/well) toward hHb (Sigma) was assessed by ELISA, using standard protocols; HRP-conjugated goat anti-human IgG antibodies (Jackson ImmunoResearch) were employed for detection.

### Hb Quantification in Murine Plasma

Hemoglobin concentration in the plasma of 4-month-old FVB and NZM mice was determined using an ELISA kit (Uscn Life Science Inc.).

### Purification and Characterization of Murine Hb, and Assessment of Splenocyte Cytokine Responses to Hb

Murine Hb was purified from the blood of 4-month-old NZB/W F1 mice by a previously described protocol (18), with modifications. Briefly, heparinized blood was mixed with cold potassium phosphate-buffered saline (PBS; 10 mM, pH 6.5) and centrifuged at 3,220 *g* at 4°C for 15 min. After cell lysis with water, the lysate was dialyzed against PBS, and then centrifuged at 1,575 *g* for 45 min. The supernatant was loaded onto an equilibrated CM-52 column (Whatman). Elution was carried out under a pH gradient [10 mM potassium phosphate buffer (pH 6.5) and 15 mM potassium phosphate buffer (pH 8.5)]. Purity of eluted Hb was assessed by HPLC and silver staining, and its identity confirmed by electrospray mass spectrometry and N-terminal sequencing. Ferric (Fe^3+^) Hb was generated by addition of an equimolar concentration of H_2_O_2_ to ferrous (Fe^2+^) Hb; conversion was verified by assessing the absorption spectrum at 200–800 nm on a spectrophometer (Shimadzu).

Splenocytes (4 × 10^6^ cells) derived from young (2-month-old) and old (8-month-old) NZM and FVB mice were incubated with Fe^2+^ Hb, Fe^3+^ Hb (0.5–5 µM), or LPS (5 µg/ml) for 24 h. After replenishment of medium, cells were rested for 24 h and then re-stimulated with PMA (50 ng/ml) and ionomycin (2 µg/ml) for 24 h. Levels of IL-6, TNF-α, IL-8 (KC), and IL-10 in supernatants were quantified by ELISA (eBiosciences).

### Determination of Anti-Hb B Cell Precursor Frequencies

Varying numbers of splenocytes from 4-month-old NZM or FVB mice were cultured in the presence or absence of 15 µg/ml LPS (from *Salmonella typhosa*; Sigma) for 7 days. Supernatants were assayed for the presence of antibodies against Hb and dsDNA (used as a positive control) by ELISA. Optical densities greater than the average of un-stimulated controls +3 SDs (for respective cell inputs) were considered indicative of the presence of antibody. Data were analyzed by Poisson analysis. Briefly, for each cell input, percentages of wells negative for the measured reactivity over the total number of wells were calculated; cell inputs resulting in a value of 37.5% were adjudged the index of the respective B cell precursor frequency.

### Hb–Self Antigen Interaction

The ability of Hb to interact with self-moieties/autoantigens was assessed by ELISA and by surface plasmon resonance (SPR).

#### ELISA

Ribonucleoprotein autoantigens (La, Sm, Ro52, Ro60, RNP68k, and RNP A; Arotec Diagnostics Limited) were dispensed into ELISA plates. Reactivity to biotinylated Fe^2+^ Hb and Fe^3+^ Hb (1 µg/ml; in the presence or absence of 20 µg/ml unlabeled Hb) was revealed by the subsequent addition of streptavidin–HRP, following standard protocols.

#### Surface Plasmon Resonance

Interaction of Fe^2+^ Hb and Fe^3+^ Hb with ribonucleoprotein autoantigens was analyzed by SPR (BIAcore 2000; GE Healthcare). Individual proteins were immobilized on a CM-4 sensor chip by standard amine coupling using 0.4 M *N*-ethyl-*N*′-(dimethylaminopropyl)-carbodiimide (EDC)/0.1 M *N*-hydroxysuccinimide (NHS). Immobilized ribonucleoproteins elicited response units (RU) ranging between 50 and 3,000. Residual activated groups were blocked using ethanolamine (1.0 M, pH 8.5). The kinetics of interaction of Hb to immobilized ribonucleoproteins were assessed at 25°C at a flow-rate of 20–30 µl/min. An activated and blocked flow cell without an immobilized ligand was used to evaluate non-specific binding. After each binding cycle, sensor chips were regenerated by NaOH (10 mM). Results were analyzed using BIAevaluation version 4.1.1 software.

#### Pull Down

CCL131 cells were incubated with RIPA Buffer (2% Triton X-100, 1% sodium deoxycholate, 0.1% sodium dodecyl sulfate, 150 mM sodium chloride, and 1 mM Tris–HCl, pH 7.8) containing a protease inhibitor cocktail (Sigma), for 20 min on ice. Cells were subjected to two freeze–thaw (FT) cycles in liquid nitrogen. Tubes were centrifuged at 16,000 *g* for 15 min at 4°C to remove debris. Protein content was estimated and 1 mg of lysate was incubated with 50 µg of Sulfo-NHS-LC-Biotin (Sulfosuccinimidyl-6-{biotinamido} hexanoate; Pierce) for 30 min. The reaction was quenched using 1 M ammonium chloride, and the lysate was dialyzed against PBS to remove free biotin.

Hemoglobin-conjugated Sepharose 4B beads (prepared by standard protocols) were incubated with biotinylated CCL131 lysate for 16 h at 4°C. Beads were washed with PBS, SDS loading dye was then added, and the mixture was heated to 100°C for 20 min in a dry bath (Biosan). Samples were centrifuged at 16,000 *g*, and supernatants were processed for SDS-PAGE and Western blot. After incubation with streptavidin–HRP, enhanced chemiluminescence was employed to reveal Hb-interacting cellular moieties.

### Assessment of the Effects of Hb, Apoptotic Blebs, and Cellular Lysate on Bone Marrow-Derived Dendritic Cells (BMDCs)

Apoptosis was induced in CCL131 cells by incubation with 0.5 µM staurosporin (Bio Basic) for 24 h. Blebs were prepared using the protocol of Fransen et al. (19). For the preparation of control FT cellular lysate, cells were re-suspended in PBS, snap-frozen by brief incubation in liquid nitrogen, and immediately thawed; the cycle was repeated three times. The lysate was centrifuged at 16,000 *g* for 15 min at 4°C to remove debris.

Bone marrow cells, isolated from the femur and tibia bones of 2-month-old NZM or FVB mice, were cultured in RPMI 1640 (Life Technologies) supplemented with 0.2% NaHCO_3_, 0.24% HEPES, 10% FCS, and 40 ng/ml GMCSF (Peprotech). On day 8, immature dendritic cells were incubated for 48 h with Fe^2+^ Hb, Fe^3+^ Hb (0.5, 2.5, or 5 µM), hemin (Sigma; 2, 10, or 20 µM), apoptotic blebs (0.5, 1, or 10 μg/ml) or freeze–thawed cellular lysate (0.5, 1, or 10 μg/ml) either individually or in specific combinations; in some instances, the following signaling inhibitors (0.05–10 µM) were employed: JNK II: JNK inhibitor II; PD98059: MEK/ERK inhibitor; SB203580: p38 inhibitor; H-89: PKA inhibitor; Ly294002: PI3K inhibitor; Stat3 VII: Stat3 inhibitor VII; CAS 285986-31-4: Stat5 inhibitor (Calbiochem).

On day 10, supernatants were retrieved for cytokine analysis (eBiosciences, R&D Systems), and cells were processed for flow cytometry. Briefly, cells were incubated with biotinylated anti-CD11c (BD Biosciences) along with individual FITC-labeled antibodies against murine CD80, CD83, CD86, or CD40 (eBiosciences) for 1 h at 4°C. Cells were then incubated with streptavidin–PE (eBiosciences) for 1 h at 4°C. Cells were re-suspended in 0.2% paraformaldehyde and run on a flow cytometer (BD FACS-Calibur). Data were analyzed using Flow Jo X software (Tree Star, Inc.).

10^5^ BMDCs derived from either NZM or FVB mice and matured in the presence of either Fe^2+^ Hb, Fe^3+^ Hb, or hemin were γ-irradiated (30 G) before coculture with 3 × 10^5^ splenocytes isolated from C57BL/6 mice. After 4 days, ^3^H-thymidine (0.5 μCi/well) was added for 18 h, and cell-incorporated radioactivity was assessed on a β-counter (MicroBeta TriLux, Perkin Elmer).

The effect of Fe^3+^ Hb on signaling intermediates was determined. BMDCs derived from NZM and FVB mice were stimulated with Fe^3+^ Hb in presence or absence of inhibitors of various signaling pathways (as described earlier) for 30 min. Cell lysates were prepared in RIPA buffer containing phosphatase and protease inhibitors. After SDS-PAGE and transfer to nitrocellulose, lysates were probed using antibodies specific to the phosphorylated forms of STAT3, AKT, p38, JNK, and STAT5; antibodies binding the moieties regardless of phosphorylation status were used as control.

### Characterization of Endogenous Anti-Hb Reactivity and Reactivities Elicited upon Hb Immunization

Sera were collected from NZM and FVB mice at monthly intervals till the age of 9 months. Antibodies adhering to the kidneys and lungs at 9 months were isolated using a previously described protocol (20), with modifications. Briefly, organs were rinsed with chilled PBS, immersed in buffer containing a protease inhibitor cocktail (Sigma), and homogenized. Homogenates were centrifuged at 12,800 *g* for 10 min at 4°C. Pellets were re-suspended in citrate-phosphate buffer (0.02 M, pH 3.2) and an incubation carried out for 2 h at room temperature. Suspensions were then centrifuged at 12,800 *g* for 10 min at 4°C. Supernatants were collected and the pH neutralized with 1 N NaOH.

Eight-week-old NZM and FVB mice were either left un-immunized, were subcutaneously immunized with 50 µg Hb emulsified in incomplete Freund’s adjuvant (IFA; Difco), or were subcutaneously injected with IFA; three injections were administered at fortnightly intervals and blood samples were collected at 3, 5, 7, and 9 weeks after the initiation of immunization; reactivity of antibodies in sera at 9 weeks after the initiation of immunization is presented. Antibodies adhering to the kidneys and lungs in IFA-immunized and Hb-immunized animals at 16 weeks after the initiation of immunization were eluted as described above. Sera from non-immunized animals and from IFA- and Hb-immunized animals was diluted (1:200) and assessed for the presence of antibodies to Hb by Western blot and for antibodies to cellular antigens on permeabilized CCL131 cells by flow cytometry, using standard protocols. Sera (diluted 1:50–1:2,000) or organ eluates (diluted 1:50–1:100) were assessed for the presence of IgG + IgM antibodies to dsDNA, Fe^2+^ Hb, Fe^3+^ Hb, Hbα, Hbβ, contiguous peptides representing the murine Hb sequence (enumerated in Table S1 in Supplementary Material), ribonucleoproteins (Ro52, RNP68K, La, Sm), and various phospholipids by ELISA. IgG (purified from the serum of immunized animals by Protein A chromatography) was employed in some experiments. Isotypes of anti-dsDNA and anti-Hb antibodies were enumerated by employing biotin-conjugated isotype-specific antibodies (BD Biosciences) and subsequent incubation with streptavidin–HRP.

### Histological Analysis

At 6 months of age (or 16 weeks after the initiation of immunization for IFA- and Hb-immunized animals) and at 9 months of age (for non-immunized animals), kidneys were fixed in buffered formalin and 4 µm sections were stained with hematoxylin and eosin and methenamine silver. Indices of glomerular damage (mesangial matrix increase, mesangial hypercellularity, basement membrane thickness and sclerosis), tubular damage (degenerative epithelium and casts), and cellular infiltration of cells were scored by a pathologist unconnected with the study in blinded protocols. Glomeruli were scored for glomerulosclerosis as: normal (an absence of sclerosis), mild sclerosis (≤25% sclerosed glomerulii), moderate sclerosis (26–49% sclerosed glomerulii), or severe sclerosis (≥50% sclerosed glomerulii).

De-paraffinization and antigen retrieval of kidney sections was carried out using standard protocols. Sections were blocked by 5% normal goat serum followed by neutralization of endogenous peroxidase activity with 3% H_2_O_2_. Sections were stained with HRP-conjugated goat anti-mouse C3 antibodies (Cappel) followed by incubation with 3,3-diaminobenzidine/tetrahydrochloride (DAB; Vector Laboratories) and counterstaining with hematoxylin. 1 mm^3^ kidney sections were fixed in 0.1 M cacodylate buffer (pH 7.3) containing 2.5% glutaraldehyde and 4% formaldehyde. Modifications to the basement membrane and the presence of deposits were enumerated by transmission electron microscopy.

### Statistical Analysis

Either the Shapiro–Wilk test or the Kolmogorov–Smirnov test was applied (depending on sample size) to assess for normal distribution of data; all data demonstrated normal distribution. Statistical analysis was carried out by the unpaired Student’s *t*-test (for comparison of two groups) and one-way ANOVA (for comparison of more than two groups and for paired data).

## Results

### Anti-Hb Reactivity in Human Sera

**Figure 1:**
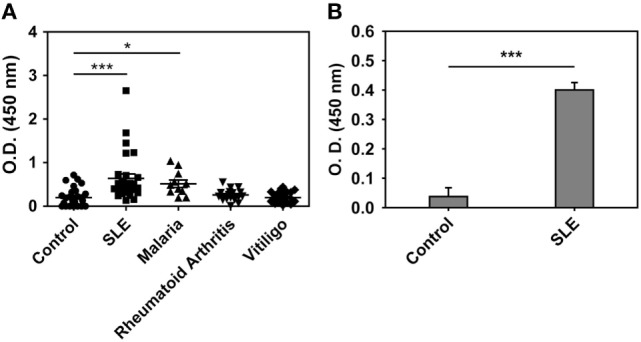
Anti-human Hb (hHb) antibodies in human sera. **(A)** Reactivity of IgG and IgM antibodies in the sera of healthy volunteers (control, *n* = 28), SLE patients (*n* = 28), malaria (*P. falciparum*) patients (*n* = 10), rheumatoid arthritis patients (*n* = 20), and vitiligo patients (*n* = 20) to hHb. Data represent mean ± SEM. **(B)** Reactivity of IgG immunoglobulins, purified from SLE sera containing antibodies reactive toward hHb (*n* = 8) and from control sera (*n* = 8), to hHb. Data represent mean ± SEM (*p < 0.05, ***p < 0.001).

Anti-hHb reactivity of IgG + IgM antibodies in the sera of SLE patients, malaria (*P. falciparum*) patients, rheumatoid arthritis patients, and vitiligo patients was assessed. Although the SLE patients constituted a distinct cross-sectional cohort, the incidence of anti-hHb reactivity was in general agreement with previous data from our lab (17); while anti-Hb antibody levels were also significantly elevated in sera of malaria patients, antibodies in sera from rheumatoid arthritis patients and vitiligo patients did not exhibit such reactivity (Figure 1A). IgG immunoglobulins were purified from the sera of eight SLE patients (demonstrating heightened anti-hHb reactivity) and eight control subjects by Protein G affinity chromatography; IgG purified from the sera of SLE patients demonstrated significantly higher reactivity toward hHb than did IgG purified from control sera (Figure 1B). Anti-Hb antibodies can therefore be detected in two diseases associated with erythrocyte lysis but not in some other diseases also characterized by inflammation and autoimmunity.

### Plasma Hb Levels, Splenocyte Cytokine Responses to Hb, Anti-Hb B Cell Precursor Frequencies, Endogenous Anti-Hb Autoantibody Reactivity

**Figure 2:**
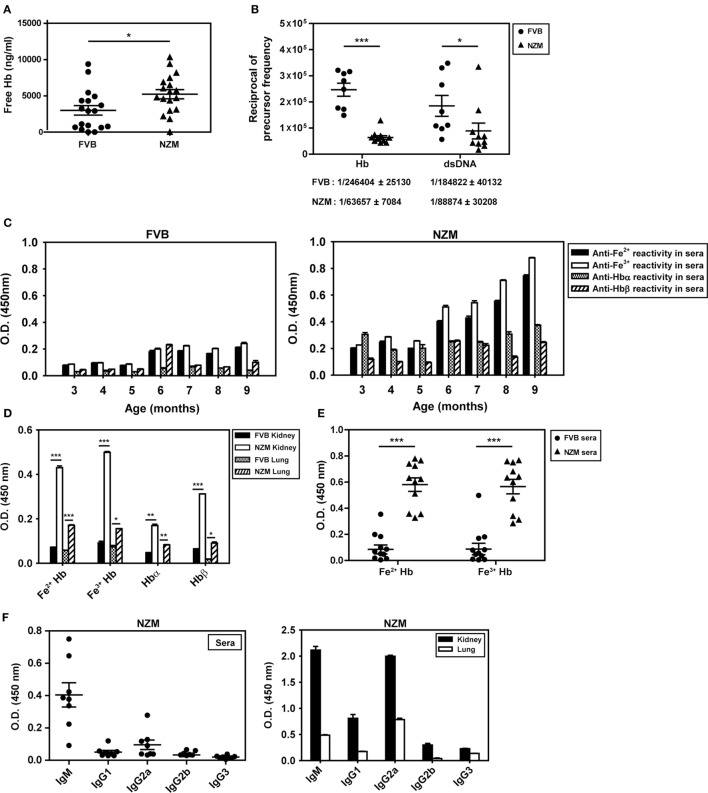
Plasma hemoglobin (Hb) levels, anti-Hb B cell precursor frequencies, and endogenous anti-Hb autoantibody responses in the sera and organ eluates of FVB and NZM mice. **(A)** Hb concentrations in the plasma of 4-month-old FVB and NZM mice (*n* = 18). Symbols represent individual mice and mean ± SEM are also depicted. **(B)** Anti-Hb and anti-dsDNA B cell precursor frequencies in 4-month-old NZM (*n* = 11) and FVB mice (*n* = 8). Symbols represent individual mice and mean precursor frequencies ± SEM are also depicted. Numerical values (mean ± SEM) of anti-Hb and anti-dsDNA B cell precursor frequencies in the two strains are indicated below each graph. **(C)** Reactivity of antibodies in pooled sera (*n* = 12) from FVB (left panel) and NZM (right panel) mice, drawn at different ages, to Hb (Fe^2+^ or Fe^3+^), Hbα, and Hbβ. **(D)** Antibodies to Hb (Fe^2+^ or Fe^3+^), Hbα, and Hbβ in kidney and lung eluates from NZM and FVB mice at 9 months. **(E)** Reactivity of antibodies in the sera of FVB and NZM mice (at 7 months) to Hb (Fe^2+^ or Fe^3+^). Symbols represent individual mice, and mean ± SEM are also depicted. **(F)** Isotype analysis of anti-Hb antibodies in sera at 7 months (left panel) and in organ eluates at 9 months (right panel) from NZM mice. Symbols in the left panel represent individual mice, and mean ± SEM are also depicted. In all bar graphs, data represent mean ± SEM (*p < 0.05, **p < 0.005, ***p < 0.001).

The concentration of free Hb in the plasma of 4-month-old lupus-prone NZM mice was higher than in the plasma of similarly aged non-lupus-prone FVB mice (Figure 2A). In addition, splenocytes derived from 8-month-old NZM mice responded to exogenous Hb (in particular, Fe^3+^ Hb) with the generation of significantly higher levels of IL-6, TNF-α, IL-8 (KC), and IL-10 than did splenocytes derived from 2-month-old NZM mice, or splenocytes derived from 2- to 8-month-old FVB mice; in particular, in the latter three groups of animals, levels of IL-6, IL-8 (KC), and IL-10 were either statistically insignificant or undetectable upon Hb stimulation, compared with cells incubated with medium. Notably, with the exception of IL-10 in young NZM mice, LPS induced statistically significant levels of the cytokines from splenocytes derived from young and old NZM mice, and from young and old FVB mice compared with cells incubated with medium (Figure S1 in Supplementary Material).

Limiting dilution analysis was employed to enumerate anti-dsDNA and anti-Hb B cell precursor frequencies in 4-month-old NZM and FVB mice. While anti-dsDNA B cell precursor frequencies were expectedly higher in NZM mice, anti-Hb precursor frequencies were also ≅4-fold higher in NZM mice than in similarly aged FVB mice (Figure 2B). These results indicate that lupus-prone mice express higher plasma Hb levels, exhibit higher splenic cytokine responses to the molecule in an age-dependent manner, and may additionally be prone to develop anti-Hb antibody reactivity. Higher titers of anti-Hb antibodies (recognizing both Fe^2+^ and Fe^3+^ forms of murine Hb) were indeed observed in the sera of lupus-prone NZM mice than in non-lupus prone FVB mice (Figure 2C). While anti-Hb titers in the sera of NZM mice demonstrated age-dependent increases, titers against Hbα and Hbβ remained relatively low across different ages, possibly suggesting conformation-dependent autoantibody recognition (Figure 2C). Higher titers of antibodies reactive to Fe^2+^ Hb and Fe^3+^ Hb were also observed in lung and kidney eluates from NZM mice than from FVB mice at 9 months (Figure 2D). More detailed analysis, carried out at multiple dilutions of sera drawn from NZM and FVB mice at 4 and 9 months, reiterated the fact that endogenous anti-Hb titers were higher in NZM mice and that these titers registered an age-dependent increase. Experiments carried out using equal amounts of IgG immunoglobulins purified from the serum of NZM and FVB mice also revealed heightened anti-Hb recognition in the former (Figure S2 in Supplementary Material).

Autoantibodies to Fe^2+^ Hb and Fe^3+^ Hb exhibited high penetrance in lupus-prone mice (Figure 2E; Figure S3 in Supplementary Material), with a predominance of the IgM isotype in sera and of the IgM and IgG2a isotypes in organ eluates (Figure 2F). Amino acids 100–119 of Hbα and 100–119 of Hbβ appeared to constitute major auto-antigenic B cell epitopes, since these peptides were preferentially bound by antibodies in the sera of lupus-prone mice (Figure S4A in Supplementary Material); in organ eluates from these mice, several other peptides were also recognized along with these dominant reactivities, but to a lesser extent (Figure S4B in Supplementary Material). Reactivity to a peptide with the same amino acid composition as Hbβ (100–119) but in scrambled sequence (referred to as Scrb 100–119) was low, suggesting that interaction was not charge dependent (Figures S4A,B in Supplementary Material).

### Effects of Hb on BMDC Maturation

**Figure 3:**
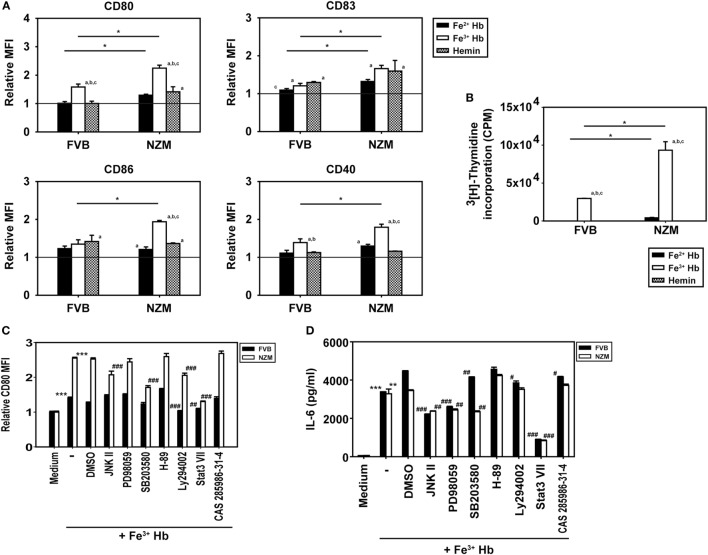
Effect of hemoglobin (Hb) on the maturation of bone marrow-derived dendritic cells (BMDCs) from 2-month-old FVB or NZM mice. **(A)** Expression of CD80, CD83, CD86, and CD40 on CD11c^+^ BMDCs stimulated with 0.5 µM Hb (Fe^2+^ or Fe^3^) or 2 µM hemin. Relative MFI: ratio of mean fluorescence intensity of stimulated over un-stimulated (or vehicle-treated) BMDCs. **(B)** Proliferation of C57BL/6 splenocytes stimulated with γ-irradiated BMDCs (derived from FVB or NZM mice) subsequent to incubation with Hb (Fe^2+^ or Fe^3+^) or hemin. **(C)** Expression of CD80 on CD11c^+^ BMDCs (derived from FVB or NZM mice) stimulated with Fe^3+^ Hb in presence of signaling inhibitors (10 µM) or the vehicle dimethyl sulfoxide (DMSO). Relative MFI: ratio of mean fluorescence intensity of stimulated over un-stimulated BMDCs. **(D)** Estimation of IL-6 in culture supernatants of BMDCs derived from FVB or NZM mice stimulated with Fe^3+^ Hb in the presence of various signaling inhibitors (10 µM) or DMSO. **(C,D)** JNK II: JNK inhibitor II; PD98059: MEK/ERK inhibitor; SB203580: p38 inhibitor; H-89: PKA inhibitor; Ly294002: PI3K inhibitor; Stat3 VII: Stat3 inhibitor VII; CAS 285986-31-4: Stat5 inhibitor. *p < 0.05, **p < 0.005, ***p < 0.001 vs medium; ^#^*p* < 0.05, ^##^*p* < 0.005, ^###^*p* < 0.001 vs DMSO. In all graphs, data represent mean ± SEM. Data derived from three independent experiments, each carried out in triplicate (*n* = 18; 9 NZM, 9 FVB).

The influence of Hb on the expression of cell surface markers on BMDCs was assessed; the gating strategy employed is depicted in Figure S5 in Supplementary Material. Fe^3+^ Hb significantly increased expression of CD80, CD83, and CD40 on CD11c^+^ BMDCs derived from FVB mice and of CD80, CD83, CD86, and CD40 on such cells derived from NZM mice, an indication of its general inflammatory potential. Fe^2+^ Hb and hemin were able to induce increases in the expression of some markers as well. While Fe^3+^ Hb induced significant up-modulation of CD80 levels compared to Fe^2+^ Hb and hemin in both mice strains, only in NZM mice were levels of CD86 significantly heightened by Fe^3+^ Hb over those achieved with either Fe^2+^ Hb or hemin. Fe^3+^ Hb enhanced levels of CD40 over those achieved by Fe^2+^ Hb in both strains of mice. Furthermore, BMDCs from lupus-prone NZM mice demonstrated more pronounced up-modulation of maturation markers in response to Hb, compared with responses observed in non-lupus prone FVB mice, while Fe^2+^ Hb induced significantly higher increases in CD80 and CD83, Fe^3+^ Hb induced significantly higher increases in CD80, CD83, CD86, and CD40 (Figure 3A). LPS was employed as a positive control in these studies; as expected, LPS induced increases in the expression of several markers on CD11c^+^ BMDCs derived from both FVB and NZM mice (Figure S6 in Supplementary Material).

While Fe^3+^ Hb-matured BMDCs derived from both FVB and NZM mice induced the proliferation of allogeneic C57BL/6 splenocytes, BMDCs derived from NZM mice were significantly better stimulators. BMDCs derived from both FVB and NZM mice matured in the presence of hemin were unable to induce proliferation (Figure 3B). Fe^3+^ Hb therefore mediated functional phenotypic and functional changes on antigen-presenting cells derived from lupus-prone mice that were distinct from those induced in non-lupus-prone mice.

Increased phosphorylation of Stat3 was observed upon the stimulation of FVB and NZM BMDCs with Fe^3+^ Hb; such increases were negated upon incubation with a Stat3 inhibitor. Phosphorylation of AKT, p38, JNK, and STAT5 remained unaltered in the presence of Fe^3+^ Hb (Figure S7 in Supplementary Material). While high basal levels of phosphorylated intermediates for some of these molecules in GMCSF-derived BMDCs may have obscured potential effects, two observations, made using a spectrum of signaling inhibitors, also revealed Stat3 to be a dominant signaling intermediate for Fe^3+^ Hb. First, inhibition of Stat3 most effectively downmodulated Hb-induced CD80 expression on BMDCs, an effect more prominently observed in cells derived from NZM mice (Figure 3C). Second, Fe^3+^ Hb-induced the secretion of IL-6 from BMDCs, and such secretion was maximally sensitive to Stat3 inhibition (Figure 3D). These results suggest that Fe^3+^ Hb enhances the phosphorylation of Stat3 in BMDCs to heighten surface expression of costimulatory markers, as well as elevates the secretion of an inflammatory, lupus-associated cytokine.

To summarize, whether in lupus-prone or healthy mice, the fact that Hb acts *via* Stat3 to elicit inflammatory responses from, and phenotypic alterations on, dendritic cells is of interest. The higher circulating levels of Hb in lupus-prone mice provide context and potential disease relevance; coupled with the heightened sensitivity to Hb in terms of the up-modulation of costimulatory molecules, these effects may be worthy of further study, given reports of dendritic cell aberrance in lupus.

### Assessment of Hb–Self Antigen Interaction and Evaluation of Downstream Consequences

**Figure 4:**
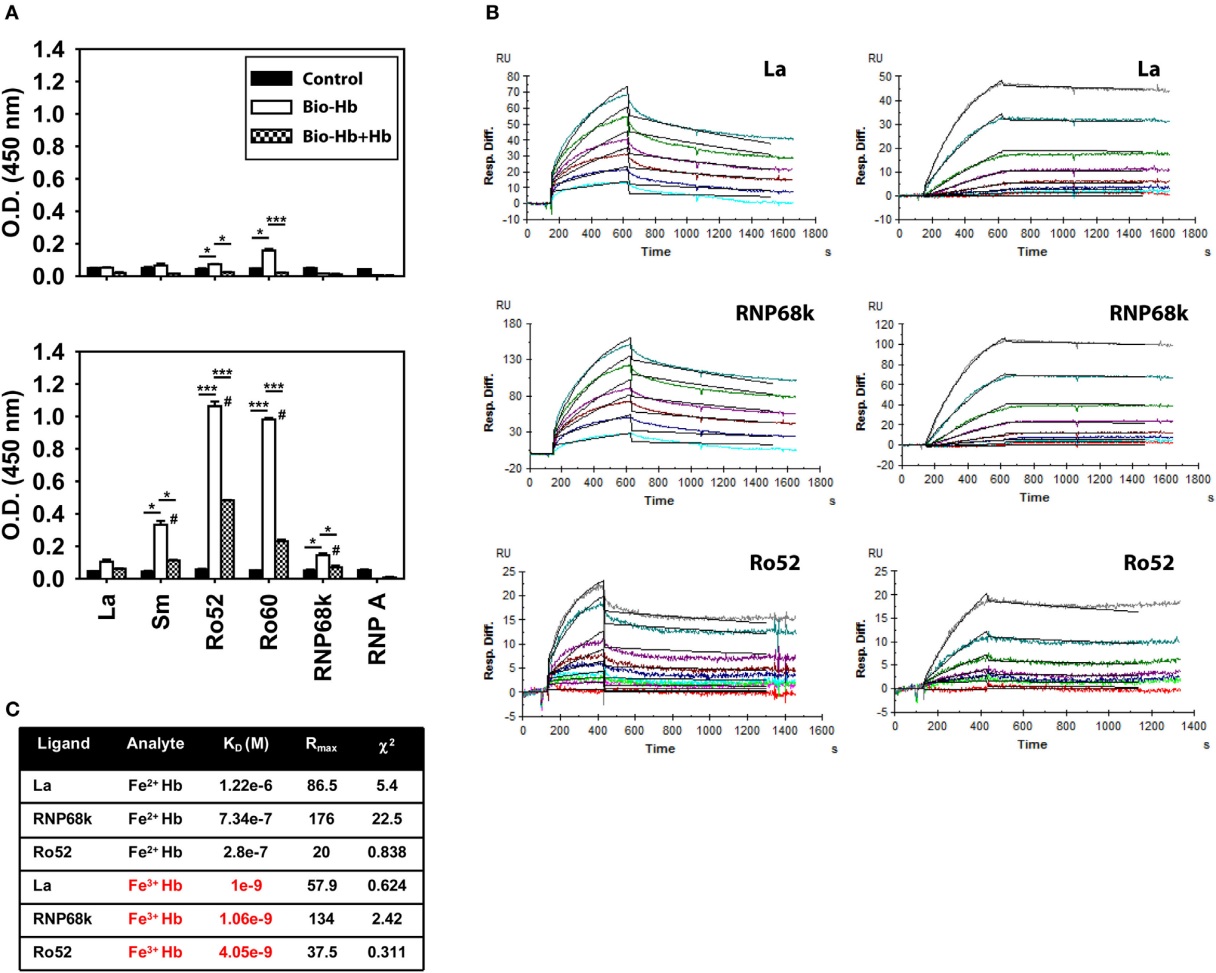
Interaction of hemoglobin (Hb) with autoantigens. **(A)** Interaction of biotinylated Fe^2+^ Hb (Bio-Hb, top panel) and biotinylated Fe^3+^ Hb (Bio-Hb, bottom panel) with the indicated autoantigens; the effects of the addition of unlabeled Hb are also shown. *p < 0.05; ***p < 0.001; ^#^*p* < 0.05 vs Fe^2+^ Hb at the corresponding concentration. **(B)** Surface plasmon resonance analysis of the binding of Fe^2+^ Hb (left panels) and Fe^3+^ Hb (right panels) with immobilized autoantigens La, RNP68k, and Ro52, as indicated. Concentrations of Fe^2+^ Hb employed to assess interaction with La and RNP68k were 0.5, 1, 2, 3, 5, and 7 µM (light blue to turquoise) and of Fe^3+^ Hb were 0, 1.56, 3.12, 6.25, 12.5, 25, 50, and 100 nM (red to gray). Concentrations of Fe^2+^ Hb employed to assess interaction with Ro52 were 0, 0.1, 0.2, 0.4, 0.8, 1.6, 3.2, 6.4, and 9.6 µM (red to gray) and of Fe^3+^ Hb were 0, 0.78, 3.12, 6.25, 12.5, 25, and 50 nM (red to gray). Black lines alongside each profile indicate data-fitting using the 1:1 (Langmuir) Binding Model. **(C)** Parameters describing the kinetics of Hb-autoantigen interaction. Data are representative of three independent experiments.

An inflammatory synergy between Hb and several exogenous TLR ligands has been documented (11). Since lupus is characterized by an aberrance in the clearance of dying cells and apoptotic blebs contain autoantigens known to act as endogenous TLR ligands [such as dsDNA, ribonucleoproteins, and HMGB1 (21)], it was of interest to assess whether Hb could interact with such molecules. In solid phase binding assays, biotinylated Fe^3+^ Hb interacted with the lupus-associated autoantigens Sm, Ro52, Ro60, and RNP68k to varying degrees; interaction was significantly diminished upon competition with unlabeled Fe^3+^ Hb (Figure 4A, bottom panel), a strong indicator of the specificity of binding. Under similar conditions, while Fe^2+^ Hb also interacted with Ro52 and Ro60 (the principal Fe^3+^ Hb-interacting moieties), levels of interaction were appreciably lower (Figure 4A, top panel). SPR, being more quantitative and sensitive, was employed to study the interaction of Hb with the ribonucleoprotein autoantigens La, RNP68k, and Ro52; much lower concentrations of Fe^3+^ Hb were required to achieve increases in RU at par with Fe^2+^ Hb (Figure 4B). Kinetic parameters, calculated using the 1:1 (Langmuir) Binding Model, indicated that Fe^3+^ Hb bound respective ribonucleoproteins with 100- to 1,000-fold higher affinity than did Fe^2+^ Hb (Figure 4C). Hb (and more particularly, Fe^3+^ Hb) can thus interact with ribonucleoprotein autoantigens at physiologically relevant affinities.

**Figure 5:**
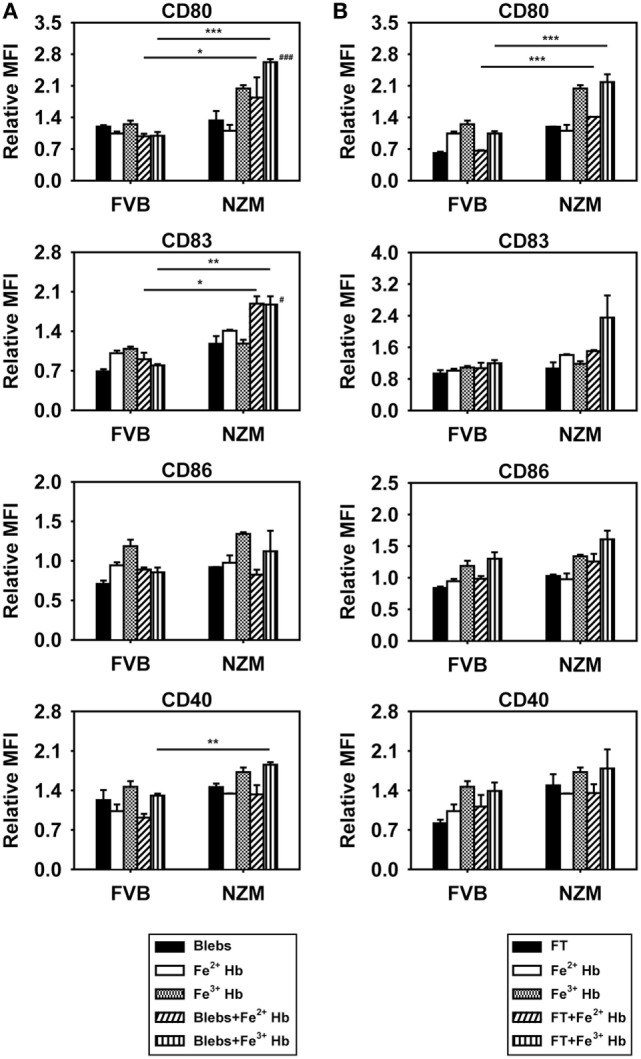
Effects of hemoglobin (Hb) and apoptotic blebs/freeze–thawed cellular lysate (FT) on maturation of bone marrow-derived dendritic cells (BMDCs) derived from 2-month-old FVB or NZM mice. Flow cytometric analysis of the expression of CD80, CD83, CD86, and CD40 on CD11c^+^ BMDCs stimulated with 0.5 mM Hb (Fe^2+^ or Fe^3+^) along with **(A)** apoptotic blebs (1 µg/ml) or **(B)** FT (1 µg/ml). Relative MFI: ratio of mean fluorescence intensity of stimulated over un-stimulated BMDCs. Data represent mean ± SEM. Differences individually attributable to Hb or blebs are not indicated. ^#^*p* < 0.05, ^###^*p* < 0.001 vs Fe^3+^ Hb and vs apoptotic blebs in the same strain; *p < 0.05, **p < 0.005, ***p < 0.001. Data derived from three independent experiments, each carried out in triplicate (*n* = 18; 9 NZM, 9 FVB).

To assess the potential consequences of Hb–autoantigen interaction, effects on the maturation of BMDCs were assessed. Expression of CD80 and CD83 was increased on the surface of CD11c^+^ BMDCs derived from NZM mice when Fe^3+^ Hb was added along with apoptotic blebs, compared to when Hb or blebs were added alone; such effects were not observed in FVB mice (Figure 5A). Coincubation of Hb with freeze–thawed cellular lysate did not result in such increases in either murine strain (Figure 5B). Furthermore, upon coincubation with Hb and apoptotic blebs, increases in CD80, CD83, and CD40 levels on BMDCs derived from NZM mice were significantly higher than on BMDCs derived from FVB mice under identical conditions. Coincubation of Fe^2+^ Hb and Fe^3+^ Hb with freeze–thawed lysate resulted in significant increases in CD80 levels in NZM- vs FVB-derived BMDCs (Figures 5A,B). These results imply that the interaction of Hb (particularly as Fe^3+^ Hb) with self-antigens (particularly as apoptotic blebs) results in immuno-stimulatory effects on dendritic cells.

### Assessment of Autoimmune Responses in Hb-Immunized Lupus-Prone and Healthy Mice

**Figure 6:**
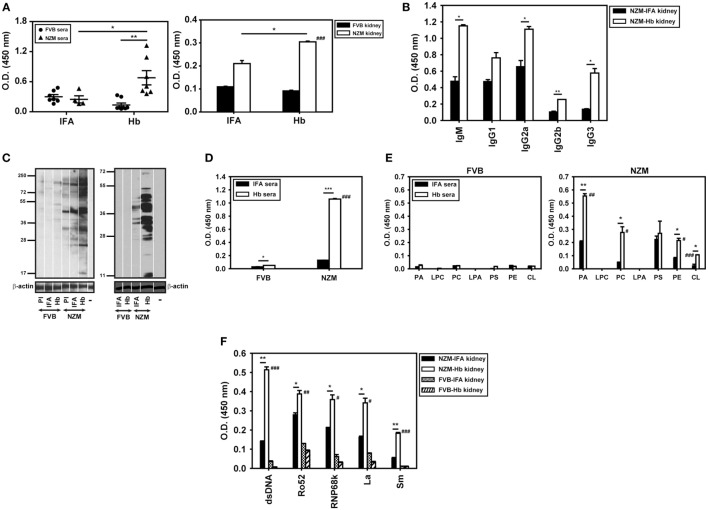
Anti-hemoglobin (Hb) autoantibody responses and autoreactivity of antibodies in sera (9 weeks after the initiation of immunization) and in kidney eluates (16 weeks after the initiation of immunization) of Hb-immunized FVB and NZM mice. **(A)** Anti-Hb antibodies in sera (left panel) and in kidney eluates (right panel) of incomplete Freund’s adjuvant (IFA)- and Hb-immunized NZM and FVB mice. Symbols in the left panel represent individual mice (*n* = 8) and mean ± SEM are also depicted. **(B)** Isotype analysis of anti-Hb antibodies in kidney eluates from Hb- and IFA-immunized NZM mice (*n* = 8). **(C)** (Left panel) Reactivity of antibodies in pooled sera (*n* = 8) from Hb- and IFA-immunized FVB and NZM mice toward moieties in cellular lysate by Western blot. (Right panel) Reactivity of antibodies in kidney eluates (*n* = 8) from Hb- and IFA-immunized FVB and NZM mice toward moieties in apoptotic blebs by Western blot. “PI,” pre-immune serum; “−,” secondary antibody control. Antibodies to β-actin were employed to verify equivalence of loading. **(D)** Anti-dsDNA antibodies in pooled sera (*n* = 8) from Hb- and IFA-immunized FVB and NZM mice. **(E)** Antibodies against phospholipids in pooled sera (*n* = 8) from Hb- and IFA-immunized FVB (left panel) and NZM (right panel) mice. **(F)** Reactivity against dsDNA and ribonucleoproteins (Ro52, RNP68K, La, Sm) of antibodies in kidney eluates (*n* = 8) of Hb- and IFA-immunized FVB and NZM mice. In all bar graphs, data represent mean ± SEM. ^#^*p* < 0.05, ^##^*p* < 0.005, ^###^*p* < 0.001 vs Hb-immunized FVB mice, *p < 0.05, **p < 0.005, ***p < 0.001.

In addition to being differentially antigenic in lupus-prone and healthy mice, whether Hb is also differentially immunogenic was assessed. Anti-Hb antibody titers increased significantly in the sera and kidney eluates of NZM mice immunized with Hb, but not in these fluids of similarly immunized FVB mice (Figure 6A). The fact that NZM mice responded to immunization with Hb by generating higher anti-Hb titers than did FVB mice was reiterated in assays carried out at multiple dilutions (Figure S2 in Supplementary Material). In addition, experiments carried out using equal amounts of IgG immunoglobulins purified from the serum of Hb-immunized NZM and FVB mice also revealed heightened recognition of Hb (beyond expected endogenous age-related increases) upon immunization of the former (Figure S2 in Supplementary Material). In kidney eluates derived from Hb-immunized NZM mice, both IgM and IgG anti-Hb antibody levels were heightened (Figure 6B). In NZM mice, anti-Hb autoantibody responses were predominantly directed against amino acids Hbα (100–119) in sera (Figure S8A in Supplementary Material) and amino acids Hbβ (100–119) in kidney eluates (Figure S8B in Supplementary Material), epitopes also targeted by endogenously arising anti-Hb autoantibody responses.

Flow cytometric analysis using permeabilized murine cells as targets revealed that Hb immunization in NZM mice, but not in FVB mice, generated antibodies that demonstrated recognition of self-moieties (Figure S9 in Supplementary Material). On Western blots upon cellular lysate and apoptotic blebs, antibodies in the sera and kidney eluates derived from Hb-immunized NZM mice recognized a wide spectrum of moieties; antibodies in the sera and kidney eluates derived from similarly immunized FVB mice were poorly reactive (Figure 6C). Antibodies against dsDNA (an antibody specificity associated with glomerulonephritis) were preferentially heightened in sera derived from Hb-immunized NZM mice (Figure 6D).

The fact that NZM mice responded to immunization with Hb by generating higher anti-dsDNA titers than did FVB mice was reiterated in assays carried out at multiple dilutions (Figure S2 in Supplementary Material). In addition, experiments carried out using equal amounts of IgG immunoglobulins purified from the serum of Hb-immunized NZM and FVB mice also revealed heightened recognition of dsDNA (beyond expected endogenous age-related increases) upon immunization of the former (Figure S2 in Supplementary Material).

Antibodies to several phospholipids were also preferentially heightened in sera derived from Hb-immunized NZM mice, as were antibodies recognizing dsDNA, Ro52, RNP68k, La, and Sm in kidney eluates from these mice; once again, equivalent increases were not observed in Hb-immunized FVB mice (Figures 6E,F). Several autoantibody isotypes contributed to increased anti-dsDNA antibody titers in sera and kidney eluates of Hb-immunized NZM mice (Figure S10 in Supplementary Material).

**Figure 7:**
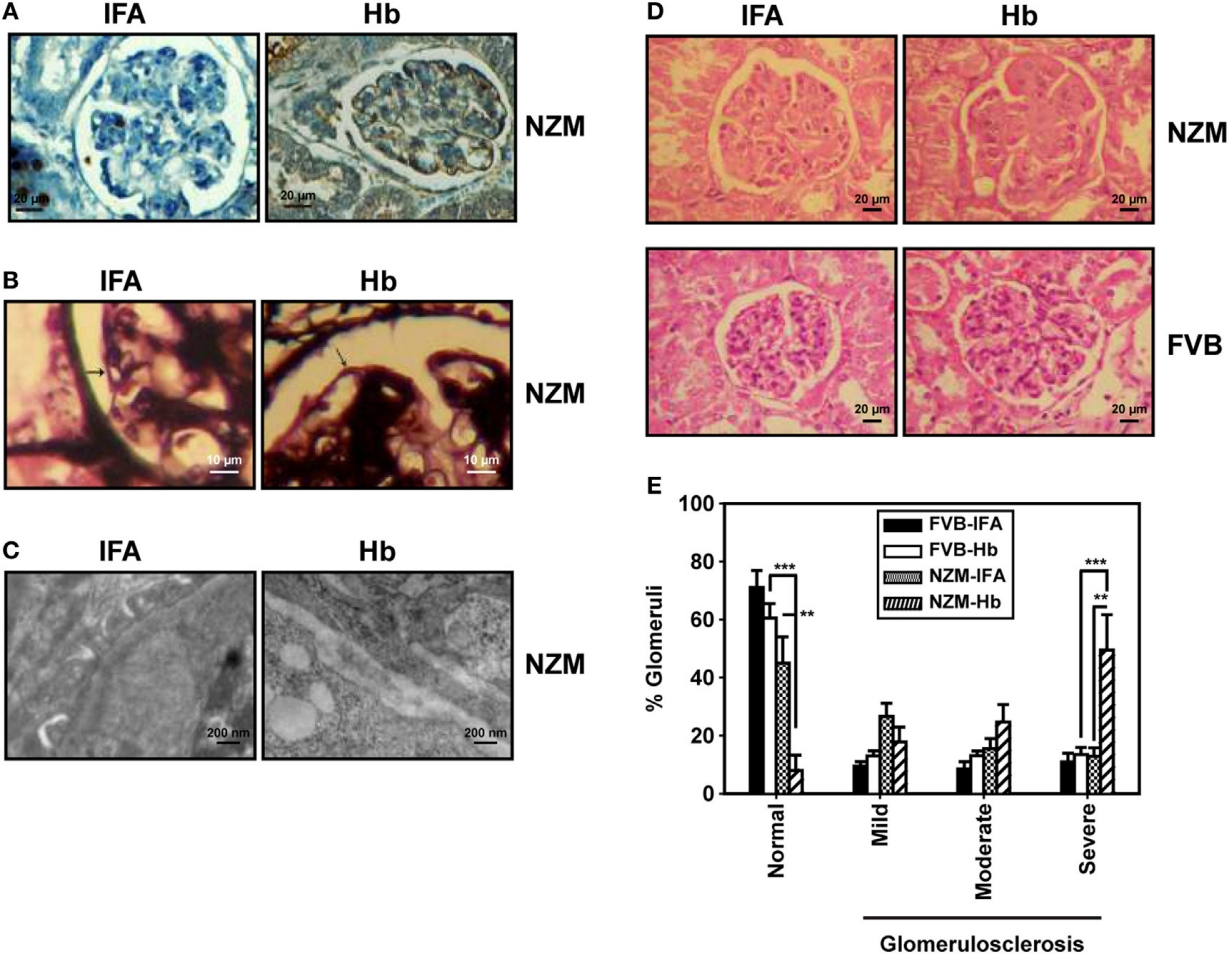
Histological analysis of kidneys from FVB and NZM mice. **(A)** Representative photomicrographs depicting reactivity of anti-C3 antibodies toward glomerulii of incomplete Freund’s adjuvant (IFA)- and hemoglobin (Hb)-immunized NZM mice 16 weeks after the initiation of immunization (40×; scale bars: 20 µm). **(B)** Representative photomicrographs depicting methenamine silver staining of glomerulii sections from IFA- and Hb- immunized NZM mice 16 weeks after the initiation of immunization. Basement membranes have been indicated by arrows (40×; scale bars: 10 µm). **(C)** Representative electron micrographs depicting kidneys from IFA- and Hb-immunized NZM mice 16 weeks after the initiation of immunization (12,000×; scale bars: 200 nm). **(D)** Representative photomicrographs depicting hematoxylin- and eosin-staining of kidney sections from IFA- and Hb-immunized FVB and NZM mice 16 weeks after the initiation of immunization (40×; scale bars: 20 µm). **(E)** Quantitative analysis (at 16 weeks after the initiation of immunization) of glomerulosclerosis (categorized as “mild,” “moderate,” or “severe”) in IFA- and Hb-immunized FVB and NZM mice (*n* = 8). Data represent mean ± SEM (**p < 0.005, ***p < 0.001).

These results suggest that, specifically in a lupus milieu, Hb can act as an early immunological trigger, inducing humoral autoreactivity toward three major classes of autoantigens (nucleic acids, ribonucleoproteins, and lipids), reactivity against each of which is associated with significant pathological sequelae in lupus. Whether glomerulonephritis, possibly the most significant of such sequelae, was indeed induced upon Hb immunization was determined. Specific deposition of complement 3 was observed along the glomerulii basement membrane in Hb-immunized NZM mice. Moreover, Hb immunization in these mice resulted in split basement membranes in the capillary loops at the edges of the glomerulii; electron photomicrographs confirmed thickening of basement membranes, accompanied by electron dense deposits. IFA-immunized NZM mice demonstrated none of these effects (Figures 7A–C). Hematoxylin- and eosin-stained kidney sections from Hb-immunized NZM mice revealed increases in the mesangial matrix and evidence of severe glomerulosclerosis without interstitial inflammation and red blood cells in the extra-capillary space; such degenerative changes were not observed in the kidneys of IFA-immunized NZM mice, or Hb- and IFA-immunized FVB mice (Figure 7D). Quantification of data revealed that a significantly higher percentage of glomerulii exhibited severe sclerosis in Hb-immunized NZM mice, compared with IFA-immunized NZM mice, and Hb- and IFA-immunized FVB mice; correspondingly, the percentage of histologically normal gomerulii was significantly diminished in Hb-immunized NZM mice (Figure 7E). The extent of glomerulosclerosis in Hb-immunized NZM mice at 6 months was equivalent to, or exceeded, that observed in naturally aging, non-immunized NZM animals at 9 months (data not shown). No significant changes in tubule-interstitial parameters were observed in these mice. Taken together, these observations constitute immunological and physiological evidence that breakage of tolerance to Hb can enhance global autoreactivity and preferentially enhance disease kinetics exclusively in a lupus-prone milieu.

## Discussion

Hematological aberrations are common in systemic lupus erythematosus (22), and anti-RBC antibodies have been demonstrated in lupus and other systemic autoimmune diseases (23). While IgG antibodies induce erythrophagocytosis *via* the Fc receptor, IgM antibodies induce erythrocyte lysis *via* activation of complement (24). Under normal physiological conditions, released Hb is efficiently cleared by the reticuloendothelial system (1). In diseases associated with excessive RBC lysis such as lupus, levels of free Hb can overwhelm scavenging mechanisms (2, 3). Hb, under such conditions, can oxidize and dissociate into dimers, and heme may be released (25). Reports suggest that Hb, oxidized Hb, Hb dimers, as well as heme can be inflammatory, toxic, redox-active, and/or vasoactive, both *in vitro* and *in vivo* (6–10).

Although T cell reactivity toward Hb (14) and anti-Hb autoantibodies (17) have been described in systemic autoimmunity, the immunobiology of Hb has not been investigated in detail. In this study, sera derived from a certain percentage of lupus patients exhibited anti-hHb humoral autoreactivity, in consonance with a previous report from our lab (17); IgG immunoglobulins purified from such sera also bound hHb. No obvious correlation was observed between autoimmune hemolytic anemia and the presence of anti-Hb antibodies. This could possibly be attributed to the fact that, while SLE patients from whom blood samples were withdrawn for this study were at various stages of disease progression, autoimmune hemolytic anemia usually occurs at disease diagnosis or in the first year of onset, with recurrence rates being low as disease progresses (26, 27). At present, it is therefore unclear whether autoimmune hemolytic anemia is solely responsible for the generation of anti-Hb antibodies, and further analysis on more patients would be instructive. It is interesting that, while anti-Hb autoreactivity was also detected in the sera of malaria patients (a disease also characterized by hemolysis), rheumatoid arthritis patients and vitiligo patients did not exhibit such antibodies. While further investigations into the causes and consequences of anti-Hb autoreactivity are warranted, what is clear that such reactivity is not the consequence of a non-specific response in all inflammatory or autoimmune diseases.

Higher concentrations of Hb were observed in the plasma of NZM mice, compared with the plasma of FVB mice. Three interesting, physiologically relevant, correlates of this phenomenon were observed. The first correlate was the heightened Hb-induced secretion of lupus-associated cytokines exclusively from splenocytes derived from old lupus-prone mice, an observation which suggests an age-related increase in sensitivity toward Hb (particularly Fe^3+^ Hb) exclusive to these animals. The fact that the non-specific inflammatory stimulus that LPS delivers did not elicit such discriminatory responses argues for an Hb-specific effect. The second correlate was the higher anti-Hb antibody-secreting B cell precursor frequencies observed in lupus-prone mice, mimicking the higher frequencies of anti-dsDNA, anti-Sm, anti-Ro, and anti-La antibody-secreting B cells seen in lupus in previous studies (28–30), an observation supporting the positioning of Hb as a *bona fide* autoantigen. The third correlate was the age-related increase in anti-Hb antibodies in NZM mice; incidence rates of reactivity were much higher in these mice than in SLE patients, possibly on account of genetic identity of the former, since immune responses are influenced by genes encoded by the major histocompatibility complex. The presence of IgG anti-Hb antibodies, particularly in organ eluates, is indicative of T cell involvement. IgG2a anti-dsDNA antibodies (among other isotypes) have been associated with glomerulonephritis (31) and may be involved in complement-mediated damage to the kidney. Whether the organ-adhered IgG2a anti-Hb autoantibodies described in this study similarly contribute to organ pathology requires further substantiation. Nevertheless, the existence of isotype-switched anti-Hb autoantibody responses in lupus-prone mice is clearly significant.

Myeloid DCs (mDCs, which help in clearance of apoptotic debris and secrete inflammatory cytokines) and plasmacytoid DCs (which secrete IFN-α and influence autoantibody generation) have been implicated in lupus pathogenesis (32). While oxidized Hb has been shown to enhance LPS-driven phenotypic and functional maturation of mDCs (33), the independent effects of Hb on mDC maturation, particularly in the context of autoimmunity, have not been previously reported. In this study, Fe^3+^ Hb was more efficient in mediating the phenotypic maturation of CD11c^+^ BMDCs derived from lupus-prone mice. Furthermore, these cells were functionally capable, inducing higher proliferative responses from allogeneic splenocytes than Hb-matured BMDCs derived from non-lupus prone mice. These findings indicate that Fe^3+^ Hb preferentially provides a biologically relevant stimulus to innate immune cells derived from lupus-prone mice. Fe^2+^ Hb and hemin were relatively less effective than Fe^3+^ Hb in mediating the observed effects. Whether the structural modifications that occur upon the oxidation of Hb (34) influence binding to putative novel cell surface receptors on BMDCs from NZM mice is currently unclear.

Various signaling pathways can contribute to DC differentiation and function (35). The fact Fe^3+^ Hb induced the phosphorylation of Stat3 in BMDCs and that inhibition of Stat3 signaling prevented the maturation-inducing effects of Hb is of interest. These observations are at variance with some previous studies which show that Stat3 negatively affects DC maturation (36–39). That said, the association of aberrant Stat3 activation and autoimmunity has also been recognized. Suppressor of cytokine signaling 1 (an inhibitor of Stat1 and Stat3 activation) deficient DCs are believed to contribute toward the generation of autoantibodies *in vivo* (40), while some of the effects of embelin (an anti-inflammatory and pro-apoptotic compound) in experimental autoimmune encephalomyelitis may be mediated by the inhibition of Stat3 in DCs (41). While oxidative stress [which is induced by Hb (6, 42, 43)] is a known activator of Stat3 phosphorylation (44, 45), the molecular events arising due to Hb-mediated activation of Stat3 in dendritic cells in the context of lupus autoimmunity deserve further attention.

Hemoglobin is known to synergize with TLR2, TLR3, TLR4, TLR7, and TLR9 agonists to elicit the secretion of IL-6 and TNF-α from macrophages (11). The addition of Hb along with HMGB1 and TLR ligands results in three-way synergy (46). Two probable mechanisms may contribute to such effects: The first mechanism postulates Hb-mediated up-modulation of TLR expression, thereby making cells more sensitive to TLR ligands. Indeed, the pro-oxidative activity of Hb generates reactive oxygen species (47) and oxidative stress has been shown to upmodulate TLR levels (48). Fe^3+^ Hb increased mRNA levels of TLR3, TLR7, TLR8, and TLR9, with concurrent enhancement in the secretion of IL-8 (data not shown). Whether such up-modulation also results in heightened TLR signaling is under investigation. The second mechanism envisages the interaction of Hb with TLR agonists to form active complexes. Apoptotic blebs contain packaged autoantigens, many of which act as endogenous TLR ligands (49) and have stimulatory effects on the maturation of BMDCs (19). The current study revealed that Hb (and more particularly Fe^3+^ Hb) interacted, at physiologically relevant affinities, with several moieties known to be present in such blebs. A more comprehensive approach to find additional potential Hb-binding partners, employing pull-down assays on cellular lysates using either Hb or mouse serum albumin (MSA, as a “control” murine protein) on the solid support, revealed that Hb interacts with a distinct set of self-moieties than does MSA (Figure S11 in Supplementary Material). The positive identification (by mass spectroscopic analysis) of at least some specific Hb-interacting moieties as autoantigens would provide further supporting evidence and is an effort that is currently on-going. The differential stimulatory effects of apoptotic blebs in combination with Hb on marker expression on BMDCs derived from lupus-prone mice are indicative of differential signaling. The fact that apoptotic blebs contain concentrated (and possibly modified/processed) autoantigens might account for their increased synergy with Hb. Hb signaling through CD163 (a receptor for Hb and the Hb–Hp complex) has been shown to activate ERK and p38 in monocytes and in monocyte-derived immature dendritic cells (50). In turn, cytokines and TLR ligands can modulate the expression of CD163 (51). Whether the synergies of Hb with external/synthetic (as seen in previous studies) or putative endogenous (as seen in this study) TLR ligands involve similar pathways remains to be elucidated.

Immunization of lupus-prone mice with Hb leads to heightened levels of autoantibodies to Hb. Two observations suggest that Hbα (100–119) and Hbβ (100–119) constitute dominant autoreactive epitopes. First, the same regions were targeted by endogenously generated anti-Hb antibodies and by antibodies generated upon Hb immunization (in both sera and organ eluates) in NZM mice. Second, anti-Hb antibodies arising in the blood of non-lupus-prone animals (at low tires) target the same epitopes; it can be postulated that these regions are inherently antigenic (and immunogenic?), perhaps due to unappreciated molecular mimicry with an unknown agent or moiety. Such a theoretical claim awaits further analysis.

While early autoimmune responses have a restricted specificity, the onset of clinical disease kinetically overlaps with the presence of autoantibodies targeting several different moieties, believed to arise as a consequence of epitope spreading and cross-reactivity (52–54). Interestingly, immunization of lupus-prone mice with Hb leads to the generation of antibodies to several other self-moieties. ELISAs reveal that kidney eluates from Hb-immunized NZM mice contain enhanced levels of antibodies to dsDNA, Ro52, RNP68k, La and Sm, and Western blots on cellular moieties, using both serum and kidney eluates, indicate a wide spectrum of reactivity toward proteinacious moieties; while reactivities possibly indicative of the recognition of Ro52 (≅52 kDa), RNP68k (≅68–70 kDa), and La (≅48 kDa) were observed, it can be speculated, based on molecular weight, that Ro60 (≅60 kDa), Sm B (≅26 kDa), and Sm D (≅13 kDa) constitute other potential reactivities. Definitive and comprehensive enumeration of all autoreactive targets awaits further analysis.

Although histological analysis carried out at 6 months indicated the enhanced onset of glomerulosclerosis specifically in Hb-immunized lupus-prone mice (compared with adjuvant-immunized mice), Figure S2 in Supplementary Material indicates anti-Hb antibodies as well anti-dsDNA antibodies were already elevated at 4 months in these mice. Investigations into whether degenerative changes in the kidneys are apparent earlier on are therefore clearly warranted.

Previous work in the lab has established that all immunizations with self-proteins in lupus-prone mice do not result in enhanced autoreactivity; while immunization with some apoptotic cell-specific antibodies in lupus-prone mice generates heightened levels of anti-self-reactivity, immunization with others does not (55). These results indicate that the effects observed upon the immunization of Hb in lupus-prone mice were not simply the consequence of non-specific inflammatory events.

**Figure 8:**
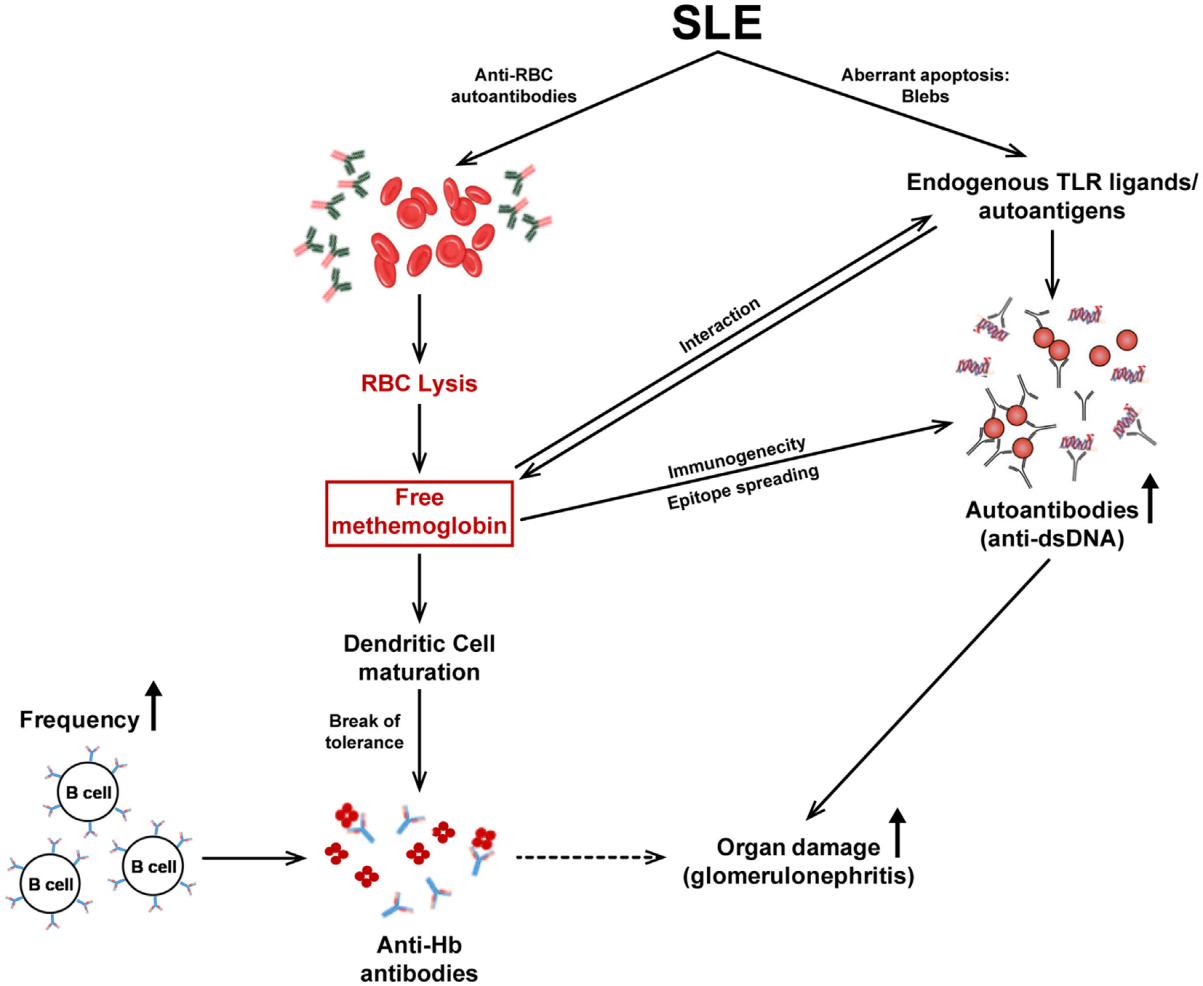
The immunobiology of Hb in systemic autoimmune disease. Autoimmune hemolysis leads to the release of Hb. If the haptoglobin-mediated clearance mechanism is overwhelmed, free Hb readily oxidizes to methemoglobin (Fe^3+^ Hb). Fe^3+^ Hb interacts with endogenous toll-like receptor (TLR) ligands/autoantigens and DAMP molecules present in apoptotic blebs, present at high concentrations in lupus. Hb and autoantigens, independently or as complexes, affect the functional maturation of antigen-presenting cells and subsequent cytokine secretion. Mature DCs present antigens to T cells leading to their activation; the ensuing cytokine secretion assists in the generation of autoantibodies by B cells. In addition, the higher Hb-specific B cell precursor frequencies in autoimmune-prone mice, as well as the binding of Hb to conventional endogenous TLR ligands/autoantigens (against which activated T cells already exist) may contribute to the break in B cell tolerance to Hb that ensues; consequently, isotype-switched anti-Hb antibodies arise in the blood. The immunogenicity of Hb is revealed (upon active immunization) to be associated with epitope spreading, possibly resulting from converse assistance to B cells specific for conventional autoantigens as a consequence of Hb–autoantigen complexation; heightened autoimmune reactivity against proteinacious, nucleic, and lipidic autoantigens results. The increased presence of autoantibodies to Hb and dsDNA (as well as to other moieties) in the kidneys correlates with the deposition of complement and with severe glomerulosclerosis.

A model elucidating the different facets of the immunobiology of Hb described in this report is depicted in Figure 8. Upon the release of Hb subsequent to autoimmune hemolysis, physiological clearance mechanisms can be overwhelmed, resulting in high circulating Hb levels. Fe^2+^ Hb can oxidize to Fe^3+^ Hb which then interacts with endogenous TLR ligands/autoantigens, available as components of apoptotic blebs. Hb and Hb–autoantigen complexes affect the functional maturation of antigen-presenting cells and splenocyte cytokine secretion in lupus-prone mice. While mature dendritic cells can then more efficiently present self-antigens to T cells, higher Hb-specific B cell precursor frequencies and higher circulating Hb levels may contribute to the specific break in B cell tolerance to Hb that specifically ensues in a lupus milieu. Hb–autoantigen complexes can also enable the recruitment of “help” from autoantigen-specific T cells to Hb-specific B cells (and *vice versa*). The breakage of tolerance to Hb is associated with epitope spreading, the sequestration of isotype-switched autoantibodies against lupus-associated proteinacious, nucleic, and lipidic autoantigens in the kidneys and the accelerated onset of glomerulosclerosis.

The current work positions Hb both as an antigen and as a pathology-inducing (or, at the very least, pathology-perpetuating) immunogen in a murine model of lupus. It exhibits the properties of an “ideal” autoantigen: It is normally sequestered, is released as a consequence of disease processes and is consequently heightened in circulation, is inherently inflammatory, is endogenously immunogenic only in a lupus milieu, and finally is disease enhancing upon immunization, again only in a lupus milieu. This is not to suggest that free Hb is solely responsible for the generation of autoimmune responses in lupus, or indeed that it has a major role, but rather that it could provide an additional stimulus in a lupus-prone environment.

While the implications of anti-Hb autoreactivity in humans remain to be determined, the possibility of the adverse effects cannot be ignored, given the murine data. The fact that anti-Hb antibodies are observed only in a certain percentage of lupus patients does not reduce their potential significance; indeed, being such a heterogeneous disease, none of the autoantibodies thus far described are seen in all lupus patients. For example, incidence rates for anti-dsDNA antibodies (an autoreactivity with possibly the highest sensitivity and specificity for lupus) can range from 30 to 90%. Regardless of this fact, specific pathologies have been attributed to specific autoantibody specificities and therefore each new specificity (such as anti-Hb) may be of consequence. Whether anti-Hb antibody levels are of supplemental diagnostic and/or prognostic value in humans would be interesting to evaluate.

## Ethics Statement

*Human subjects*: This study was carried out in accordance with the recommendations of the ethical guidelines for biomedical research on human participants laid down by the Indian Council of Medical Research with written informed consent from all subjects. All subjects gave written informed consent in accordance with the Declaration of Helsinki. The protocol was approved by the Institutional Human Ethics Committee of the National Institute of Immunology. *Animal subjects*: This study was carried out in accordance with the recommendations of Committee for the Purpose of Control and Supervision of Experiments on Animals (CPCSEA). The protocol was approved by the Institutional Animal Ethics Committee (IAEC Number: 323/13) of the National Institute of Immunology.

## Author Contributions

SJ and RP designed the study. SJ, AB, HS, and RS performed research and collected data. BB and AJ acquired and analyzed electron microscopy data. SJ and RP analyzed and interpreted data, performed statistical analysis, and wrote the manuscript. All authors were involved in drafting the work, revising it critically for important intellectual content, approved the final version to be published, and agreed to be accountable for the content of the work.

## Conflict of Interest Statement

This research was conducted in the absence of any commercial or financial relationships that could be construed as a potential conflict of interest.
