# Supplementary material for: Oxidized Hemoglobin Is Antigenic and Immunogenic in Lupus
Source: Front Immunol. 2017 Jun 26;8:732. doi: 10.3389/fimmu.2017.00732 (PMC5483465; doi:10.3389/fimmu.2017.00732)
Supplement: Supplementary file 1 [file Presentation_1.PDF]

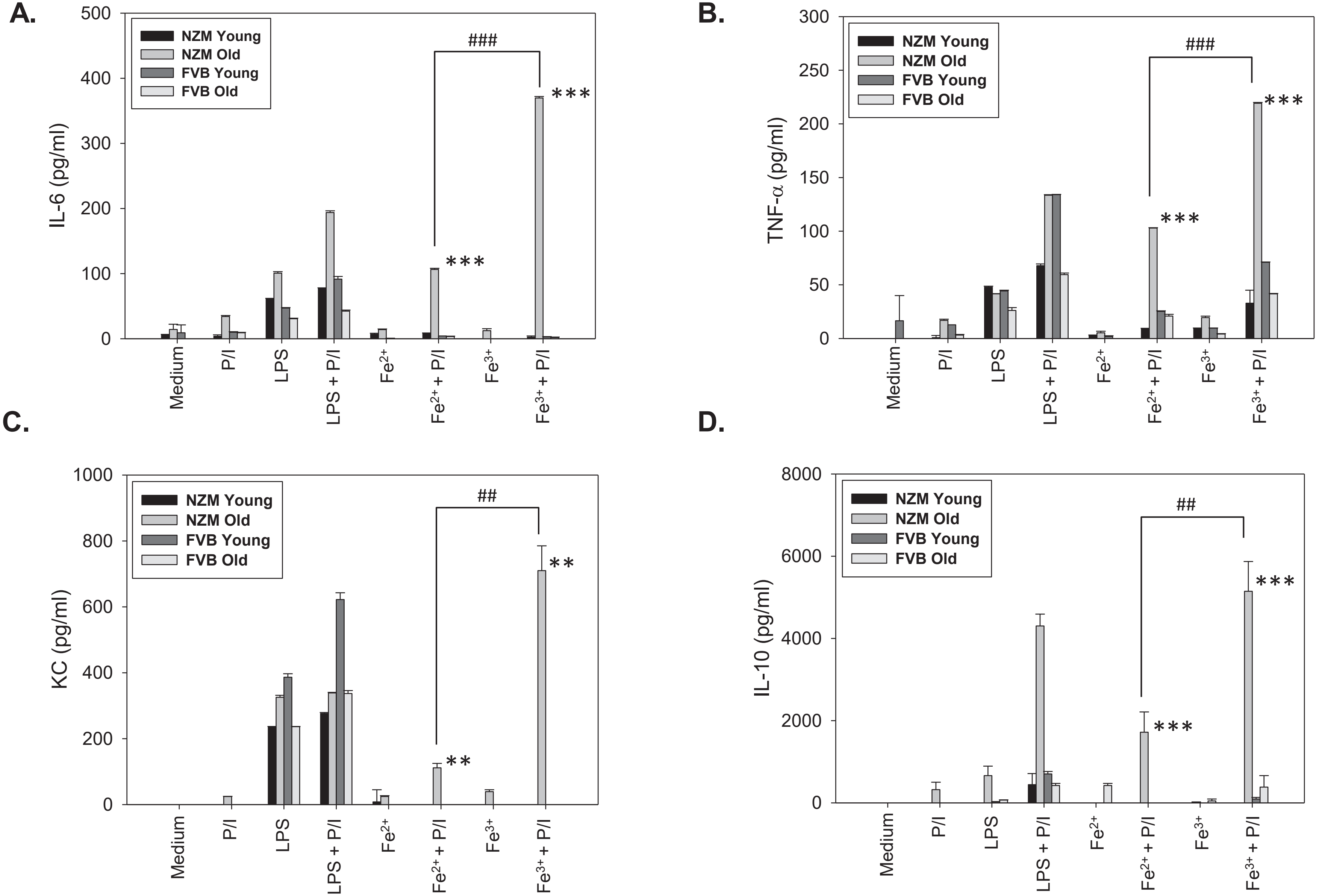

Supplementary Figure S1: Hb-induced generation of cytokines from NZM and FVB splenocytes (A) IL-6, (B) TNF-α, (C) IL-8 (KC) and (D) IL-10 levels in supernatants of splenocytes from young (2 month-old) and old (8 month-old) NZM and FVB mice incubated with 5 μg/ml LPS, 0.5 μM Fe^{2+} Hb or 0.5 μM Fe^{3+} Hb and re-stimulated with PMA plus ionomycin (P/I). Effects of individual incubations are also shown. **$p<0.01$, ***$p<0.001$ vs all other groups; $^{\#\#}p<0.01$, $^{\#\#\#}p<0.001$. $p<0.05$ for LPS + P/I vs medium for all cytokines and groups, except IL-10 in young NZM mice.

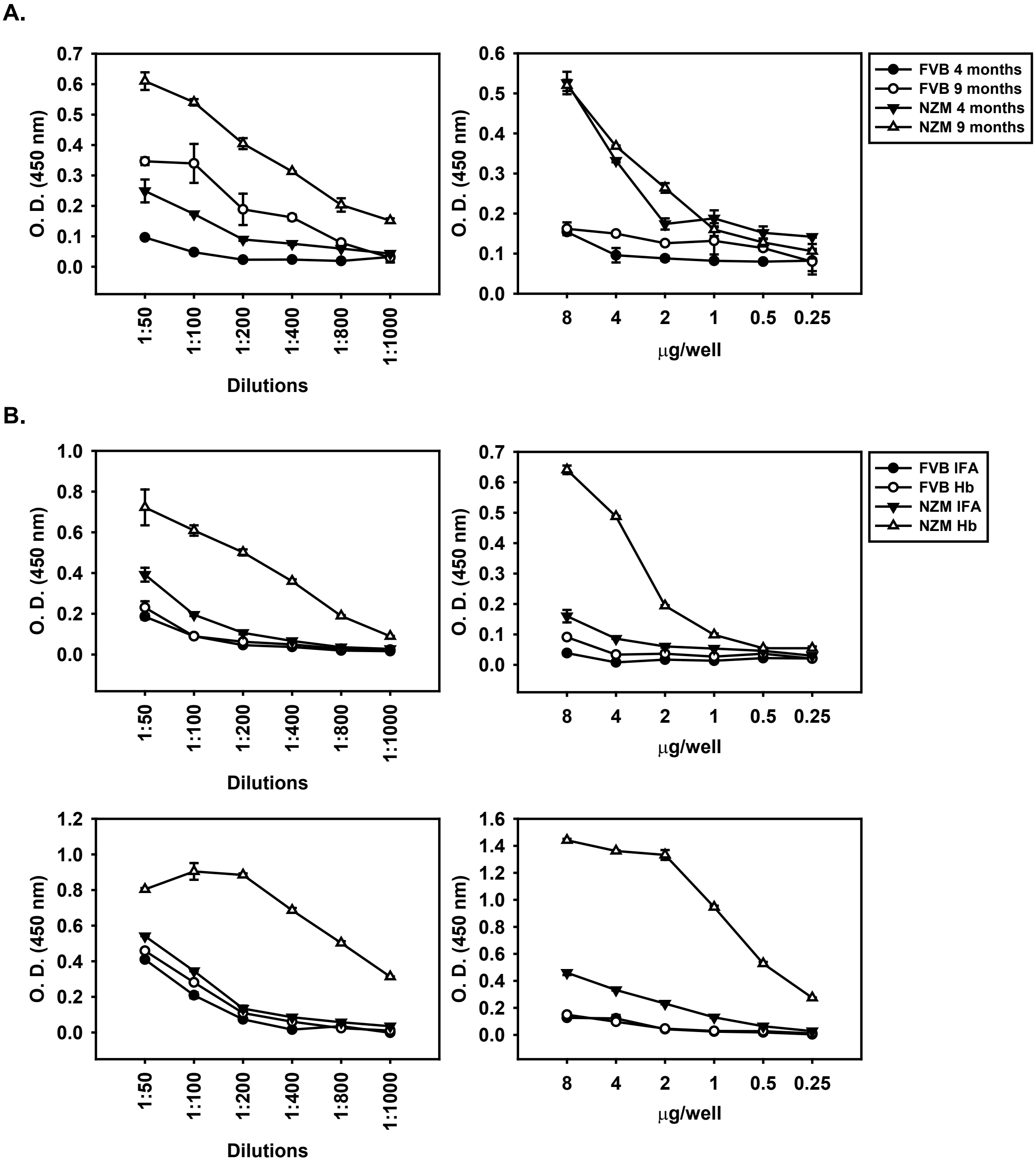

Supplementary Figure S2: Reactivity of antibodies in sera from FVB and NZM mice towards Hb and dsDNA. (A) Endogenously-arising anti-Hb reactivity at 4 months and 9 months as a function of serum dilutions (Left panel) and serum IgG concentration (Right panel). (B) Anti-Hb (Top panels) and anti-dsDNA (Bottom panels) reactivity (at 4 months) of antibodies in IFA- or Hb-immunized animals as a function of serum dilutions (Left panels) and serum IgG concentration (Right panels).

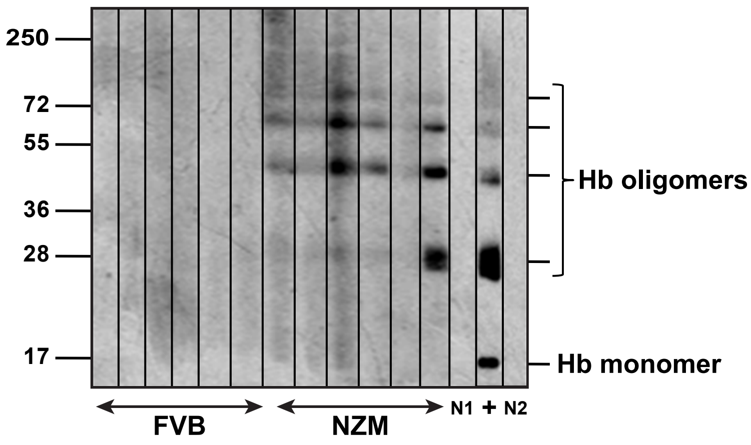

Supplementary Figure S3: Anti-Hb antibodies in the sera of individual FVB and NZM mice. Reactivity of antibodies in the sera (at 7 months) of individual FVB and NZM mice to Hb by Western blot. “N1”: anti-mouse secondary antibody control; “+”: positive control (rabbit anti-Hb antisera); “N2”: anti-rabbit secondary antibody control.

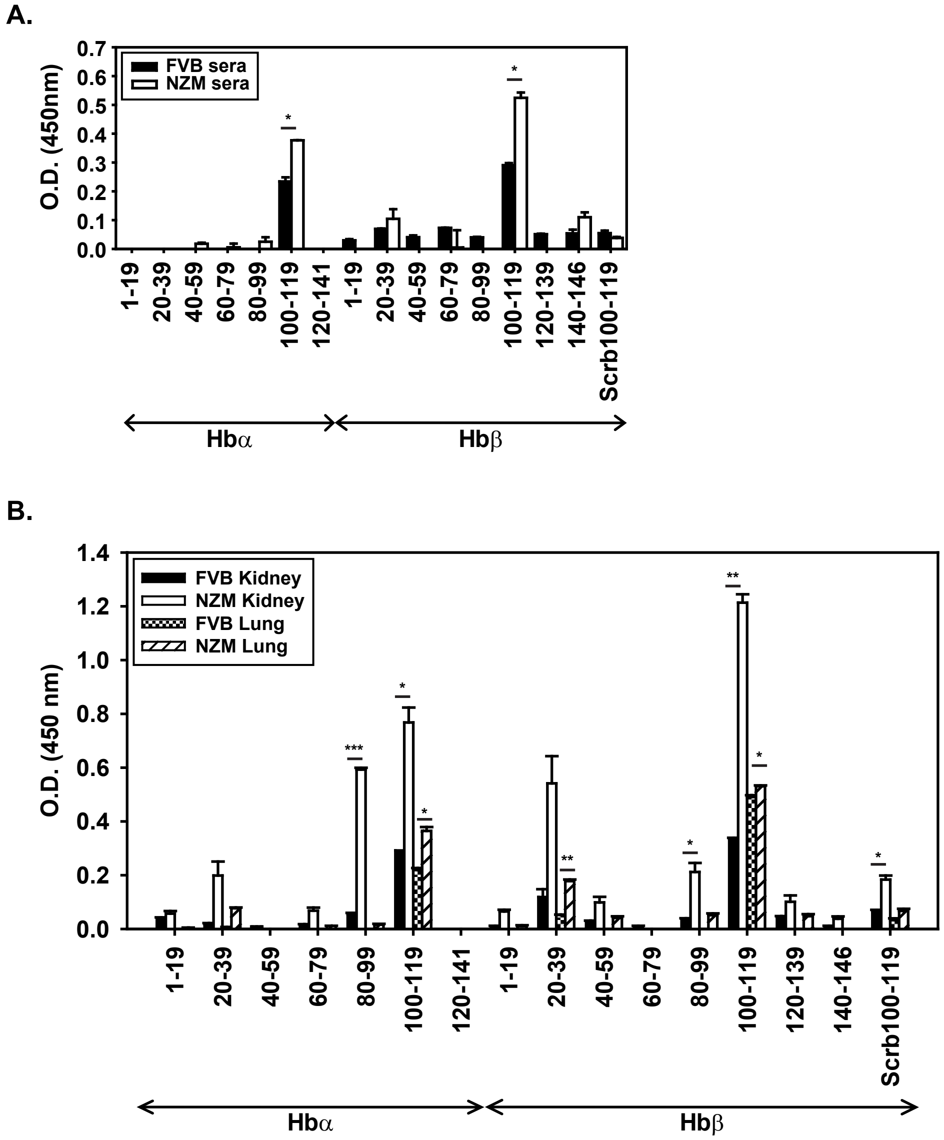

Supplementary Figure S4: Endogenous anti-Hb reactivity in FVB and NZM mice. (A) Reactivity of antibodies in pooled sera (n=12) from FVB and NZM mice (at 7 months) against contiguous peptides representing the sequences of murine Hbα and Hbβ. (B) Reactivity of antibodies in kidney and lung eluates (at 9 months) from FVB and NZM mice against contiguous peptides representing the sequences of murine Hbα and Hbβ. Scrb 100-119 refers to a peptide with the same amino acid composition as Hbβ (100-119) but in scrambled sequence. Data represents mean ± SEM. *p<0.05, **p<0.005, ***p<0.001.

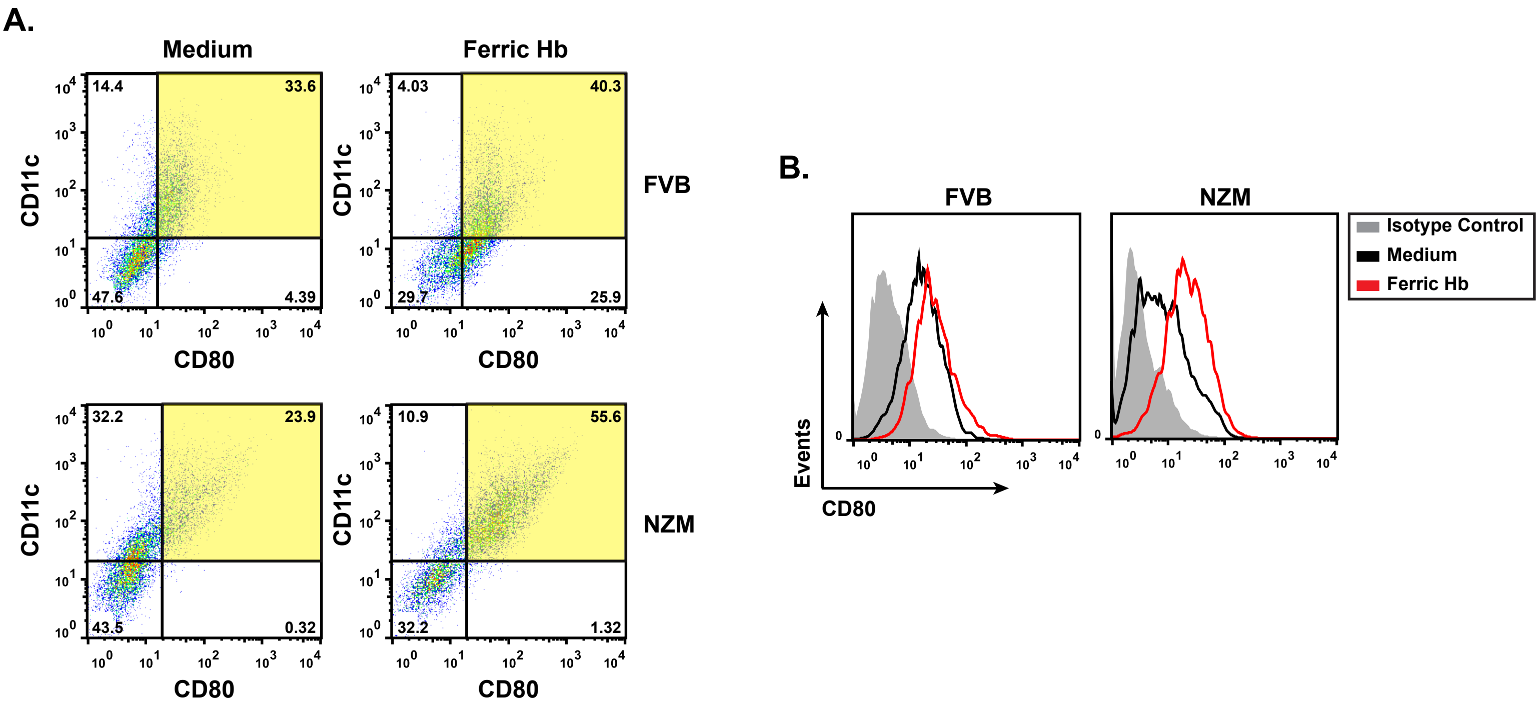

Supplementary Figure S5: Representative gating strategy for flow cytometric analysis on BMDCs derived from 2 month-old FVB or NZM mice. (A) Dot plots for CD80 and CD11c on BMDCs stimulated with 0.5 µM Fe^{3+} Hb. (B) Histograms generated by gating areas shaded in yellow in (A). MFI determinations were made from such data, which is representative of three experiments.

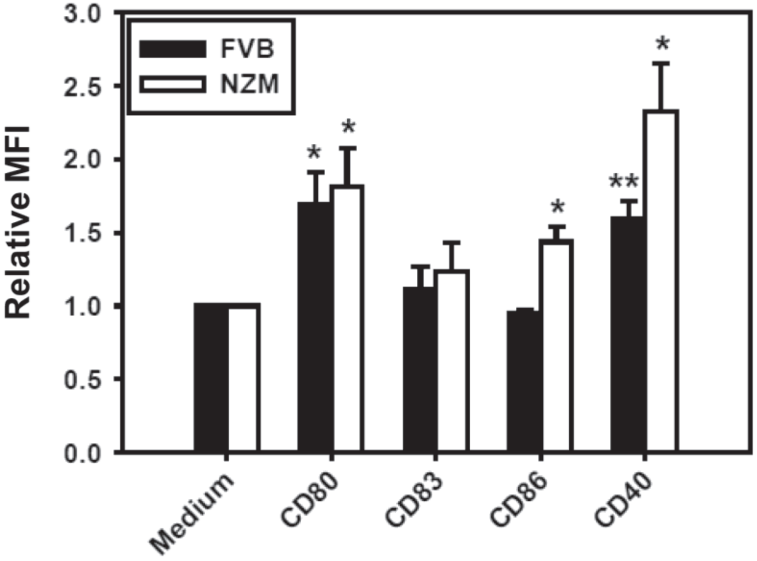

Supplementary Figure S6: Effect of LPS phenotypic markers on CD11c⁺ BMDCs derived from FVB and NZM mice. Expression of markers CD80, CD83, CD86 and CD40 on CD11c⁺ BMDCs stimulated with LPS for 48 hours. Relative MFI: Ratio of Mean Fluorescence Intensity of stimulated over un-stimulated BMDCs. Data represents mean ± SEM. *p<0.05, **p<0.005 vs medium.

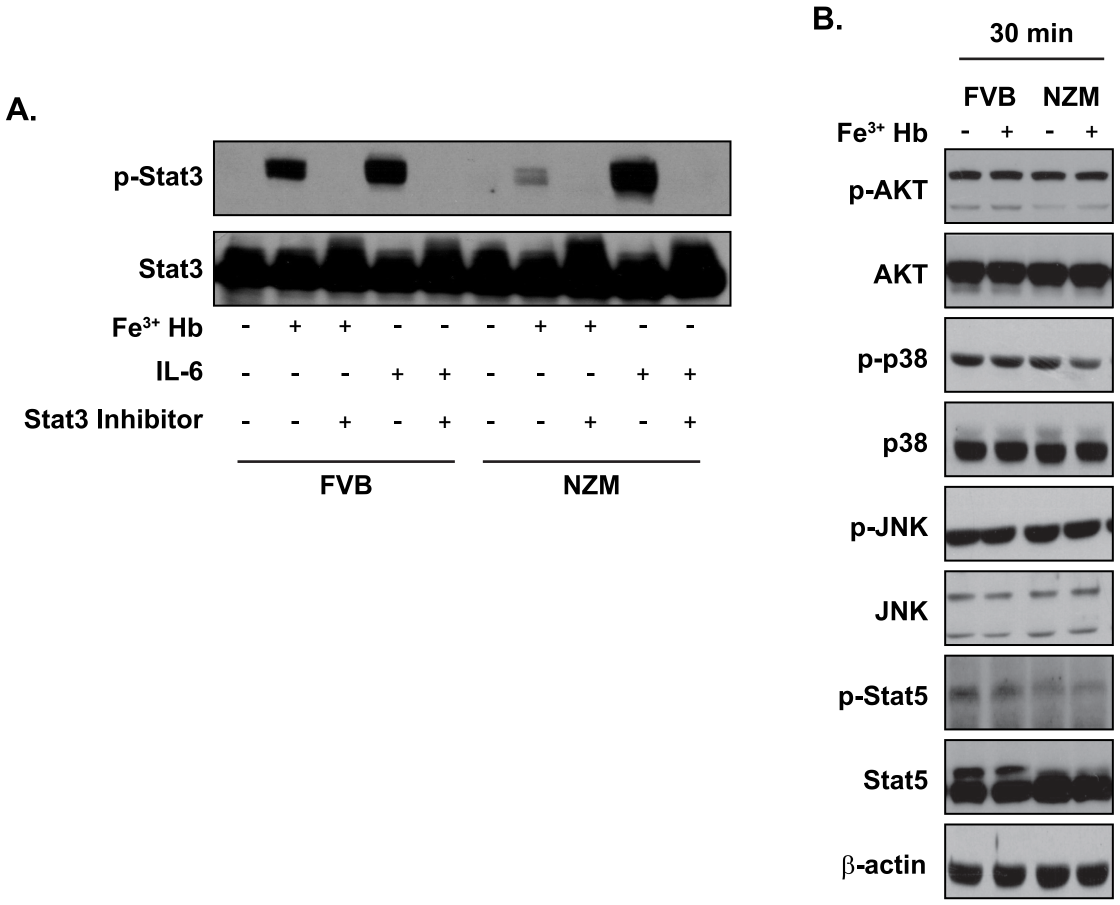

Supplementary Figure S7: Hb signaling in BMDCs derived from 2 month-old FVB and NZM mice. (A) Phosphorylated (p) and total Stat3 after stimulation of BMDCs with 0.5 µM Fe^{3+} Hb for 30 min in presence or absence of Stat3 inhibitor. IL-6 was used as positive control. (B) Phosphorylated (p) and total AKT, p38, JNK and Stat5 after stimulation of BMDCs with 0.5 µM Fe^{3+} Hb for 30 min. β-actin was used as an additional loading control. Data is representative of three independent experiments.

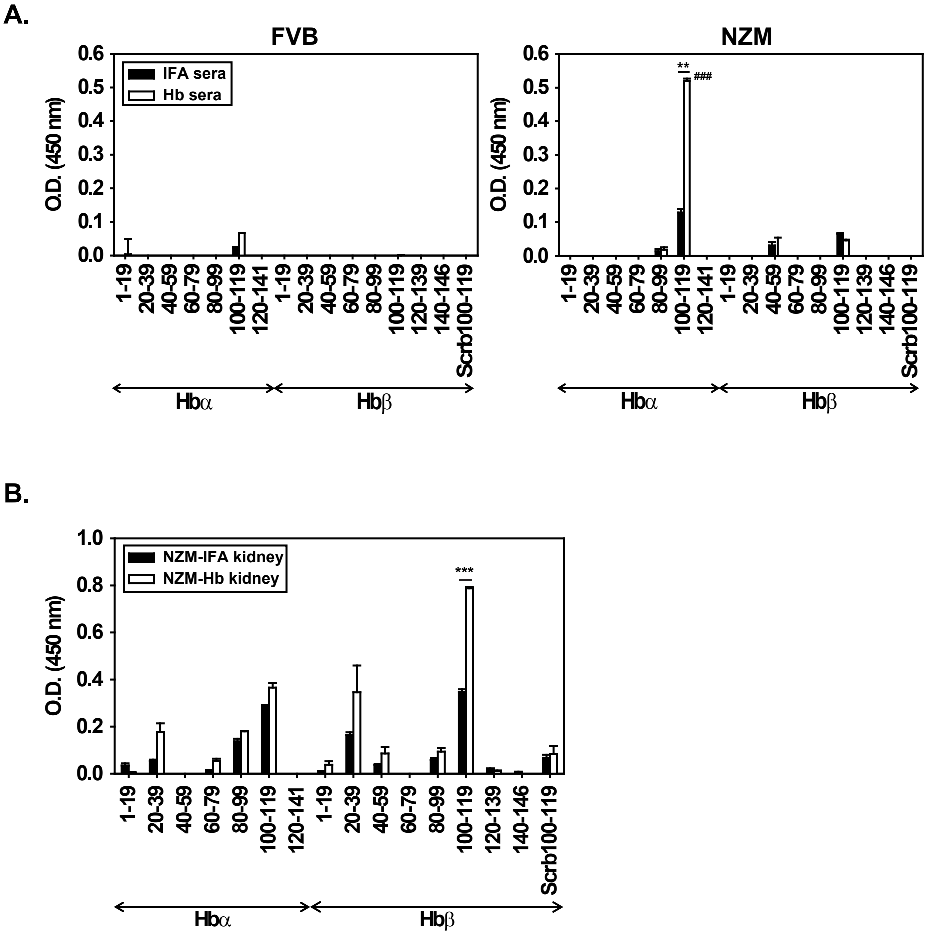

Supplementary Figure S8: Anti-Hb autoantibody responses sera (9 weeks after the initiation of immunization) and in kidney eluates (16 weeks after the initiation of immunization) of Hb-immunized FVB and NZM mice. (A) Reactivity of antibodies in pooled sera (n=8), from FVB (left panel) and NZM (right panel) mice immunized with IFA or Hb, to contiguous peptides representing the sequences of murine Hbα and Hbβ. (B) Reactivity of antibodies in kidney eluates from IFA- or Hb-immunized NZM mice (n=8) against contiguous peptides representing the sequences of murine Hbα and Hbβ. Scrb 100-119 refers to a peptide with the same amino acid composition as Hbβ (100-119) but in scrambled sequence. Data represents mean ± SEM. ###p<0.001 vs Hb-immunized FVB mice, **p<0.005.

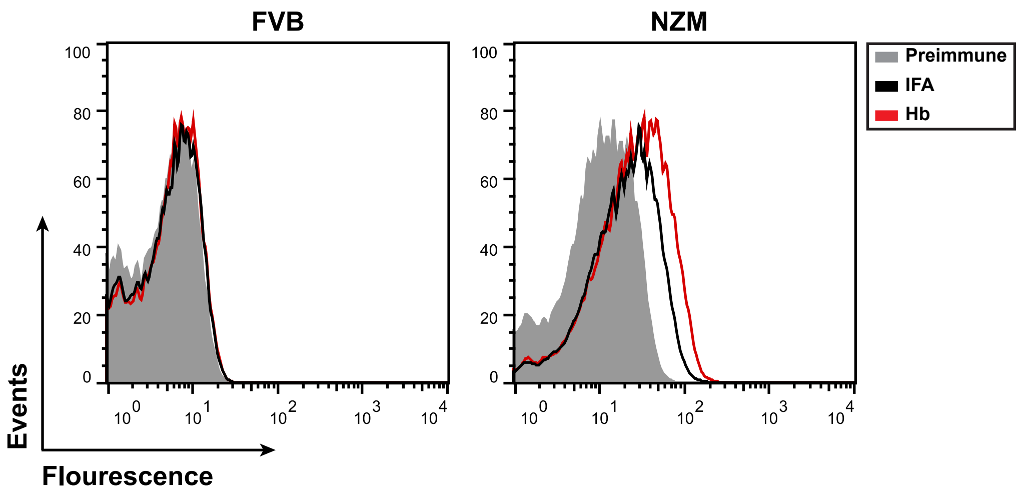

Supplementary Figure S9: Autoreactivity of antibodies in sera from IFA- and Hb-immunized FVB and NZM mice. Reactivity of antibodies in pooled sera (n=8) from Hb- and IFA-immunized FVB and NZM mice (at 9 weeks after the initiation of immunization) towards CCL131 cells by flow cytometry. Reactivity of preimmune sera is also shown. Data is representative of three independent experiments.

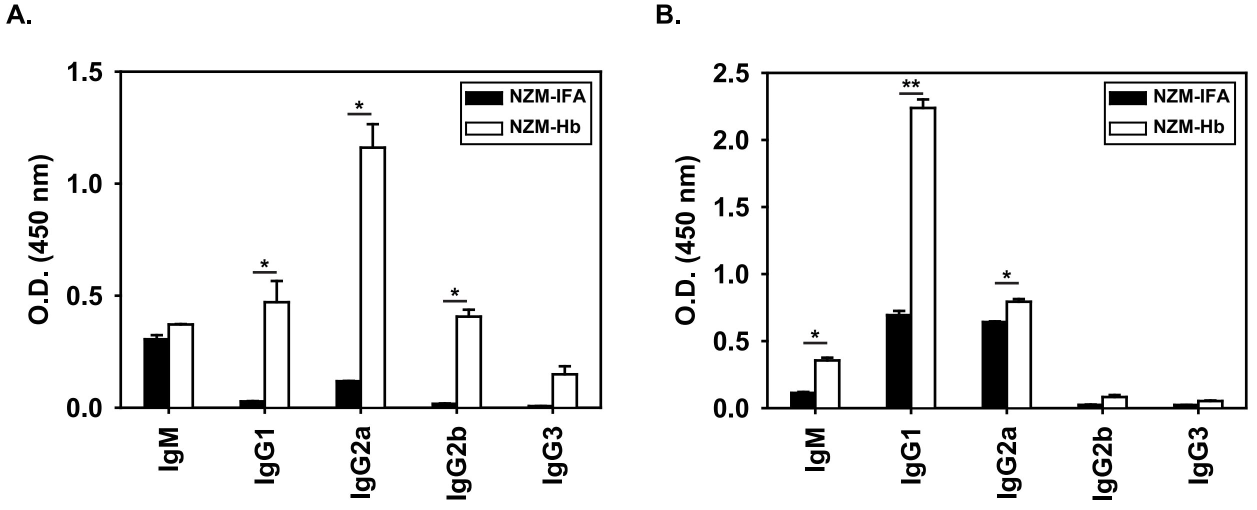

Supplementary Figure S10: Reactivity of antibodies from IFA- and Hb-immunized NZM mice towards dsDNA. Isotype analysis of anti-dsDNA antibodies (at 9 weeks after the initiation of immunization) in (A) pooled sera (n=8) and in (B) kidney eluates (n=6) from Hb- and IFA-immunized NZM mice. Data represents mean ± SEM. *p<0.05, **p<0.005.

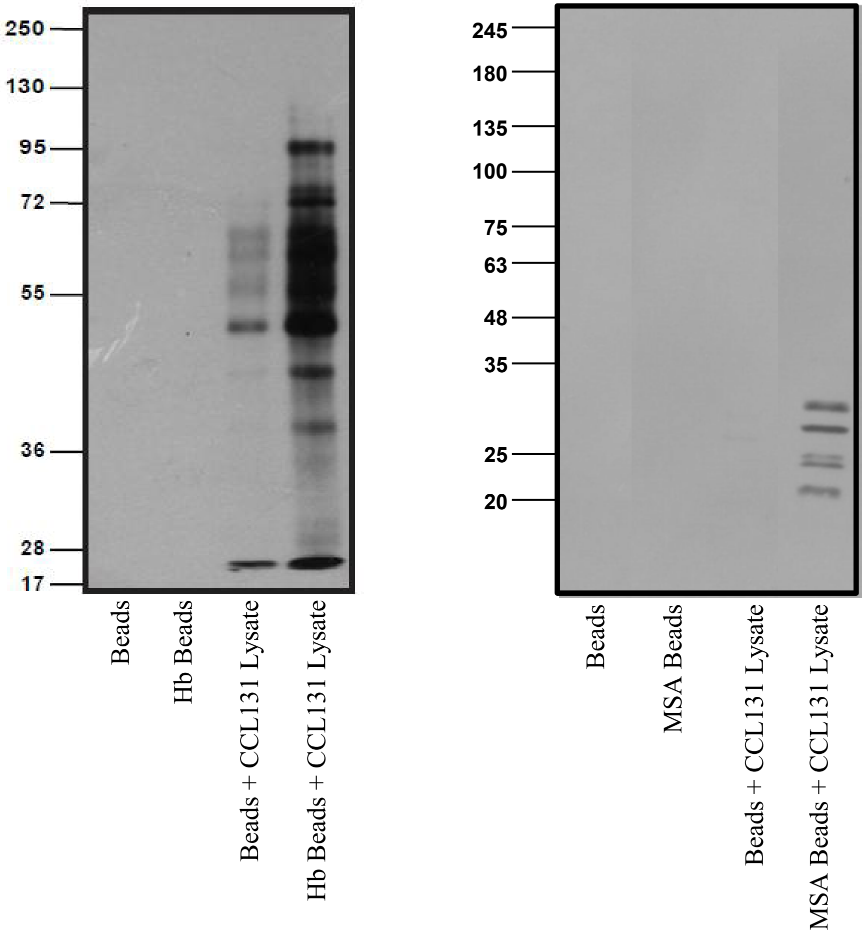

Supplementary Figure S11: Assessment of the interaction of Hb and mouse serum albumin (MSA) with cellular moieties by pull-down analysis. Moieties in biotinylated cellular lysate interacting with Hb-coupled Sepharose beads or MSA-coupled Sepharose beads were revealed by streptavidin-HRP on Western blot. Data is representative of two independent experiments.

Supplementary Table S1: Sequences of peptides representing α and β subunits of mouse hemoglobin. GenBank accession numbers based on which the peptides are derived are also shown.

Hb Subunit Acc. Number	Peptide	Sequence
Hbα Mouse:NP_001077424.1	1-19	VLSGEDKSNIKAAWGKIGG
	20-39	HGAEYGAEALERMFASFPTT
	40-59	KTYFPHFDVSHGSAQVKGHG
	60-79	KKVADALASAAGHLDDLPGA
	80-99	LSALSDLHAH KLRVDPVNFK
	100-119	LLSHCLLVTLASHHPADFTP
	120-141	AVHASLDKFLASVSTVLTSKYR
Hbβ Mouse:NP_001265090.1	1-19	VHLTDAEKAAVSCLWGKVN
	20-39	SDEVGGEALGRLLVVYPWTQ
	40-59	RYFDSFGDLSSASAIMGNAK
	60-79	VKAHGKKVITAFNDGLNHLD
	80-99	SLKGTFASLSELHCDKLHVD
	100-119	PENFRLLGNMIVIVLGHHLG
	120-139	KDFTPAAQAAFQKVVAGVAT
	140-146	ALAHKYH
	Scrb 100-119	ELIMVPLGRLGNGFHHILVN
